# ON‐OFF Switching of Photocatalytic Hydrogen Evolution by Built‐in Pt‐Nitrogen‐Carbon Reticular Heterojunctions

**DOI:** 10.1002/cssc.202401977

**Published:** 2024-11-14

**Authors:** Leonardo Cognigni, Thomas Gobbato, Elisabetta Benazzi, Lorenzo Paoloni, Biagio Di Vizio, Ruggero Bonetto, Francesco Rigodanza, Alessandro Bonetto, Stefano Agnoli, Marcella Bonchio, Paolo Costa

**Affiliations:** ^1^ Department of Chemical Sciences University of Padova Via Marzolo 1 Padova Italy; ^2^ Department of Physics and Astronomy University of Padova, Padova Via Marzolo 8 35131 Padova Italy; ^3^ Department of Environmental Sciences, Informatics and Statistics University Ca' Foscari Venice Via Torino 155, Mestre Venice Italy; ^4^ Interuniversity Consortium on Materials Science and Technology INSTM UdR Padova and Institute of Membrane Technology, ITM-CNR UoS Padova Via Marzolo 1 Padova Italy

**Keywords:** Photocatalysis, Covalent organic frameworks, N-doping, Hydrogen evolution

## Abstract

COF engineering with a built‐in, high concentration of defined N‐doped sites overcomes the “black‐box” drawback of conventional trial‐and‐error N‐doping methods (used in polymeric carbon nitride and graphene), that hamper a directed evolution of functional carbon interfaces based on structure‐reactivity guidelines. The cutting‐edge challenge is to dissect the many complex and interdependent functions that originate from reticular N‐doping, including modification of the material optoelectronics, band alignments, interfacial contacts and co‐localization of active‐sites, producing a multiple‐set of effectors that can all play a role to regulate photocatalysis. Herein, an ON‐OFF gated photocatalytic H_2_ evolution (PHE) is dictated by the Pt‐N_Pyridine_‐carbon active sites and probed with a dual COF platform, based on stable β‐ketoenamine connectivities made of triformylphloroglucinol (Tp) as the acceptor knots and 1,4‐diaminonaphtalene (Naph) or 5,8‐diaminoisoquinoline (IsoQ) as donors. Our results showcase two novel **COF‐Naph‐Tp** and **COF‐IsoQ‐Tp** frameworks featuring quasi‐identical slip‐stacked microporous structure, and similar surface area, band gap, light harvesting envelope up to 700 nm, fluorescence emission profile/lifetime, and PEIS response at the surface/water interface (R_ct_=16–10±4 KΩ). A divergent behaviour is indeed observed for **COF‐IsoQ‐Tp** with record photoelectrochemical outputs (J=−16 μA cm^−2^, R_t_=3 KΩ at 0.40 V vs RHE) and two orders of magnitude higher rate of PHE (11.3 mmol g^−1^ h^−1^, λ>400 nm, pH 5) compared to the inactive **COF‐Naph‐Tp** analogue. It turns out that PHE is regulated by the isoquinoline residues at the COF pores where emergent Pt‐N_Pyridine_‐carbon functional heterojunctions are formed upon photo‐deposition of Pt nanoparticles as co‐catalysts, as probed by combined XPS and DFT calculations evidence. This work sets a key guideline to direct the design of carbon‐based materials encoding the installation of metal‐nitrogen‐carbon active sites within tailored coordination environments enabling the catalytic performance.

## Graphical abstract

Precision N‐doping in covalent organic frameworks (COFs) enables the control over interfacial active sites while posing a formidable challenge to dissect structure‐reactivity guidelines as a way to escape the “black box” of trial‐and‐error conventional methods. Herein, an ON‐OFF gated photocatalytic H_2_ evolution (PHE) is dictated by the Pt‐NPyridine‐carbon active sites and probed with a dual COF platform, based on stable β‐ketoenamine connectivities made of triformylphloroglucinol (Tp) as the acceptor knots and 1,4‐diaminonaphtalene (Naph) or 5,8‐diaminoisoquinoline (IsoQ) as donors.

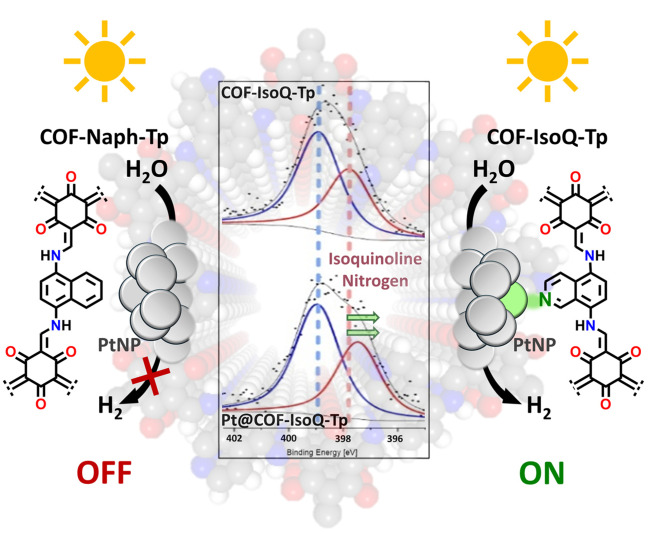

## Introduction

The incorporation of nitrogen, phosphorus, sulfur, and boron heteroatoms within carbon‐based frameworks can leverage photo(electro)catalysis for key applications related to renewable energy as water splitting and green hydrogen production.^[1]^ In particular, the insertion of nitrogen heteroatoms (N‐doping) within a carbon‐based matrix is effective to modulate the materials physicochemical properties including surface area, porosity, optoelectronics, band alignments, interfacial contacts and co‐localization of active‐sites.[^2^, ^3^, ^4^, ^5^]

With respect to the all‐carbon connectivity, N‐doping enhances visible‐light absorption by extending the π‐conjugated system, improving light harvesting, tuning redox potentials, favoring charge separation and transport over recombination.^[6]^ At the same time, N‐doping acts as non‐innocent structural effector, shaping surface and porosity properties together with N‐rich docking sites for metal‐based co‐factors, facilitating the catalytic events.^[7]^ This results in a complex blend of key enabling effectors with intertwined impact on photocatalysis and elusive *a‐solo* contributions, which poses a formidable challenge to draw relevant structure‐reactivity guidelines with predictive regulation of the final performance. Moreover, N‐doping of carbon‐based matrices lead to diverse C−N bonding modes including graphitic, pyridinic, pyrrolic, and pyridine C−N‐oxides residues, each one possessing its peculiar electronic configuration and ability to bind and interact with metal clusters, hence making it hard to discern the specific catalytic function of each C−N bonding arrangement.^[8]^ Thus, in order to control N‐doping as catalytic effector, it is essential to fine‐regulate the nitrogen type ligation‐mode, its positioning and distribution within the carbon network. On the contrary, nitrogen incorporation in several carbon materials spanning from carbon fibers, carbon nanotubes to graphene oxide and carbon nitrides relies on either (i) thermal degradation of N‐containing precursors or (ii) post‐modification of the carbon matrices by thermal treatment with N‐containing small molecules (e. g., ammonia, urea, etc.).^[9]^ In this way, efficient N‐doping is optimized by trial‐and‐error iterations, abdicating a precision chemistry control to engineer the carbon‐bonding network. Precision N‐doping is readily achieved in covalent organic frameworks (COF), i. e. porous and highly crystalline materials, whose composition, periodic structure and multi‐layer stacking are obtained by reversible covalent bond formation, thus enabling a controlled N‐doping of the carbon‐matrix based on a bottom‐up choice of the molecular precursors.[^10^, ^11^, ^12^]

This synthetic strategy has been previously adopted to tune precision N‐doping of covalent triazine‐based frameworks (CTF) by controlled poly‐condensation of tailored bipyridine (Bpy) building blocks, yielding a series of four CTF‐Bpy periodic copolymers with distinct N content and positioning of the C−N doped sites.^[13]^ The CTF‐Bpy platform provides a clear evidence of concurrent structural and photophysical phenomena, originating from N‐doping, whereby the optimized photocatalytic H_2_ evolution (PHE) results from a complex balance of the material properties, leveling off the H_2_ evolution in the range 7.2–3.2 mmol g^−1^ h^−1^ for the whole CTF‐Bpy series.

In general, the direct interaction of the N‐doped sites with the H_2_ evolving metal cocatalysts remains elusive, while a specific tuning of the metal‐nitrogen‐carbon active sites is expected to be crucial to unlock effective catalysis.[^14^, ^15^, ^16^]

**Scheme 1:**
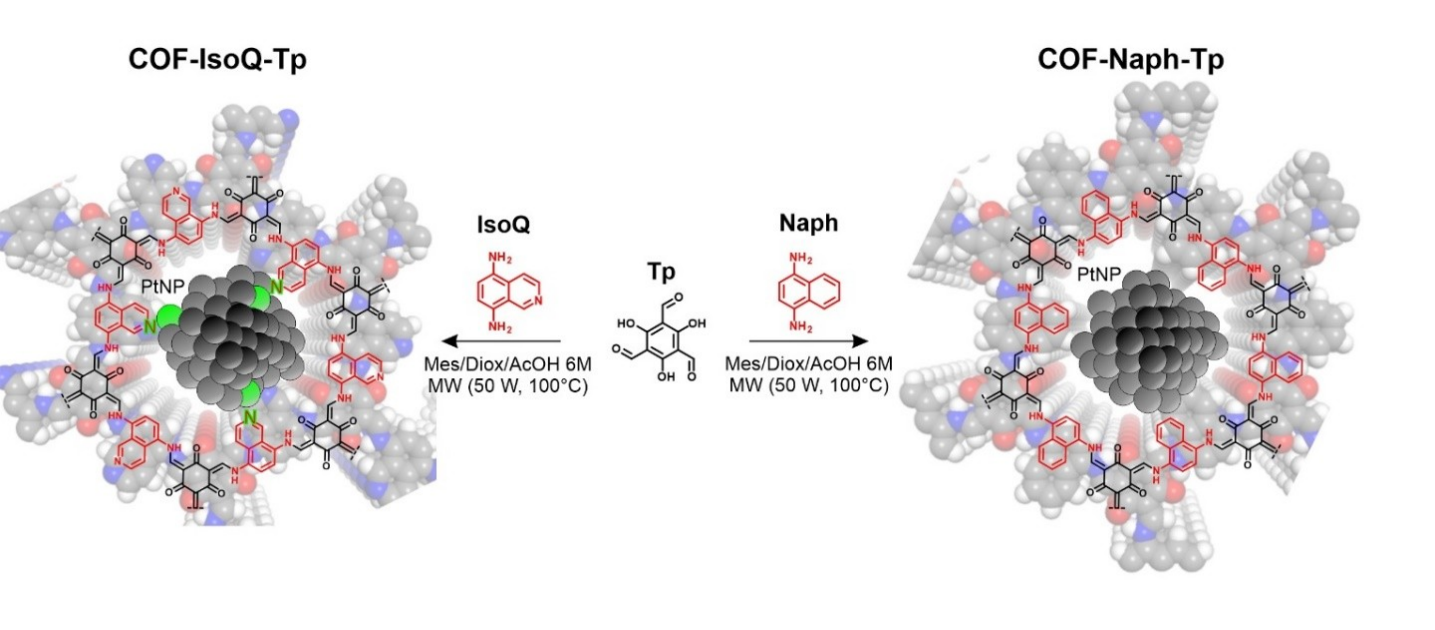
MW‐assisted reaction scheme for the synthesis of isostructural **COF‐IsoQ‐Tp** and **COF‐Naph‐Tp** under microwave‐assisted conditions and representative co‐localization of PtNPs photo‐nucleation sites at the COF pores (0.6–2.0 nm). The establishment of a functional *Pt‐N_Pyridine_‐carbon* heterojunction at the isoquinoline domains is highlighted in green for **COF‐IsoQ‐Tp** triggering photocatalytic hydrogen evolution.

Herein, we report on a dual COF platform based on stable β‐ketoenamine reticular bonding,[^17^, ^18^, ^19^] synthesized by the condensation of 2,4,6‐triformylphloroglucinol (Tp) with 1,4‐diaminonaphtalene (Naph), or 5,8‐diaminoisoquinoline (IsoQ) building blocks. Our approach points at pyridinic‐like nitrogen‐doped sites, encoded by the isoquinoline molecular synthons, and regularly positioned within the resulting **COF‐IsoQ‐Tp** framework so to be probed for PHE against the isostructural, non‐doped **COF‐Naph‐Tp** reference (Scheme 1). It turns out that despite a remarkable overlapping of both structural and optoelectronic properties, only the N‐doped **COF‐IsoQ‐Tp** provides peak photoelectrochemical activity (J=−16 μA cm^−2^, at 0.40 V vs RHE) leading to ca. two orders of magnitude higher H_2_ evolution rates (up to 11.3 mmol g^−1^ h^−1^, λ>400 nm, pH 5) compared to the inactive **COF‐Naph‐Tp** analogue. Convergent experimental and computational evidence delineates a unique scenario where the dominant N‐doping effector, probed with operando photoelectrochemical impedance spectroscopy (PEIS) and photoinduced H_2_ evolution, is responsible for a twofold activation synergy combining:

- *an enhanced charge carrier transport occurring at lower resistance in bulk **COF‐IsoQ‐Tp** compared to **COF‐Naph‐Tp** (R*_t_ *=3±1 KΩ, versus 21±2 KΩ respectively) in contrast with a similar charge transfer behavior at the surface/water interface (R*_ct_=*16±4 KΩ and 10±4 KΩ respectively)*;
- *the establishing of Pt‐N_Pyridine_‐carbon reticular heterojunctions formed upon photo‐deposition of Pt nanoparticles and co‐localized with the isoquinoline domains as probed by XPS and DFT analysis (Scheme* 1 *)*.

Our results indicate that PHE is ON‐OFF regulated by the isoquinoline residues at the COF pores, outperforming state‐of‐the art similar frameworks by ca 35 % (ca. 8 mmol g^−1^ h^−1^ SI, Table S1).[^20^, ^21^]

## Results and Discussion

**COF Synthesis and Characterization**. **COF‐Naph‐Tp** was synthesized from triformylphloroglucinol (**Tp**) and 1,4‐diaminonaphtalene (**Naph**), while **COF‐IsoQ‐Tp** has been prepared by replacing the **Naph** linker with 5,8‐diaminoisoquinoline (**IsoQ**) (SI, Section S2). Both COFs were firstly synthesized under solvothermal conditions (120 °C for five days) employing a 1 : 1 mixture of mesitylene (Mes) and 1,4‐dioxane (Diox) in the presence of 6 M aqueous acetic acid. Analogous results are readily obtained under microwave‐assisted synthesis conditions (1 : 1 mixed Mesitylene/Dioxane, 100 °C, 50 W, 90 min, Scheme 1).^[22]^ The successful formation of the β‐ketoenamine linkage is confirmed by FT‐IR analysis showing the conversion of the building blocks reactive sites (N−H and C=O signals at ca. 3300 and 1639 cm^−1^_)_ into −C=C− and C−N reticular bonds with the appearance of new bands at 1584 and 1596 cm^−1^, and at 1258 and 1251 cm^−1^, respectively for **COF‐Naph‐Tp** and **COF‐IsoQ‐Tp** (SI, Figure S1). The resulting materials elemental analysis provided the expected C/N molar ratio of 4.3 and 6.9, for **COF‐IsoQ‐Tp** and **COF‐Naph‐Tp** respectively, thus confirming the introduction of reticular N_*Pyridine*_‐carbon precision doping within **COF‐IsoQ‐Tp** (Table S2).

**Figure 1:**
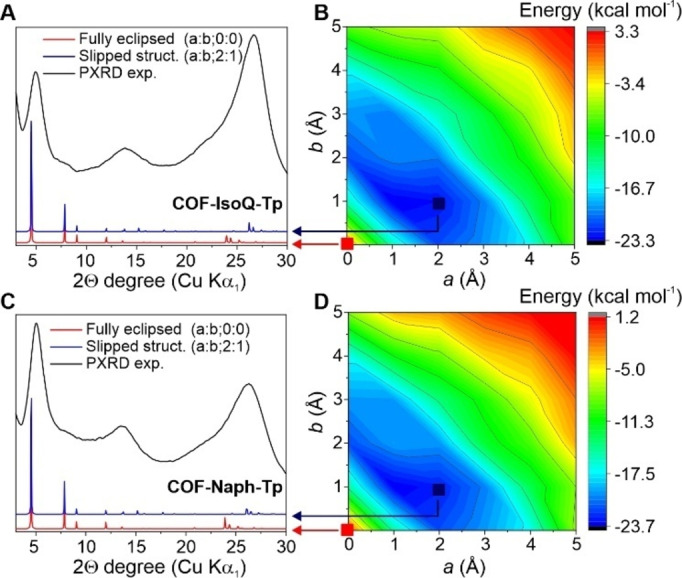
PXRD characterization and energy variation of **COF‐IsoQ‐Tp** and **COF‐Naph‐Tp** as a function of COF interlayer slipping. Experimental PXRD patterns (black line) alongside simulated patterns of the fully eclipsed COF structure (red line) and the most stable slipped‐stacked COF structure (blue line) for (A) **COF‐IsoQ‐Tp** and (C) **COF‐Naph‐Tp** respectively. The most stable structure refers to COF interlayer slippage along a and b directions by 1 and 2 Å, respectively. Contour plots illustrating the energy variation of (B) **COF‐IsoQ‐Tp** and (D) **COF‐Naph‐Tp** as a function of COF interlayer slipping along a and b directions. Energy values are referenced to the fully eclipsed structure (0.0 kcal mol^−1^), with the colour scale ranging from less stable (red region) to most stable structures (blue region).

The COF crystallinity was analyzed by powder X‐ray diffraction (PXRD, Figure 1), where both **COF‐Naph‐Tp** and **COF‐IsoQ‐Tp** show similar patterns with three broad reflexes centered at 2θ=5.02/4.93; 13.6/13.8 and 26.3/26.8 respectively. By comparison with the simulated PXRD patterns of a fully eclipsed COF structure (Figure 1A–C red lines, Figure S2), the reflexes observed at low angles (2θ<6.0°) are ascribed to the {100} plane (Pawley refinement parameters, **COF‐IsoQ‐Tp**: R_p_=1.94 %, R_wp_=2.53 %; **COF‐Naph‐Tp**: R_p_=1.35 %, R_wp_=1.59 %). Nevertheless, this model does not fully reproduce the observed broad signals at high angles (2θ>10.0°) therefore, with the help of plane‐wave DFT calculations (details in SI, Section S4), several structures of the COF unit cell have been computed as a function of the layers slipping along *a* and *b* directions.

Contour plots (Figure 1B–D) show a low energy blue region for the most favorable structures, calculated for **COF‐IsoQ‐Tp** and **COF‐Naph‐Tp** and deviating from the fully eclipsed AA stacking, with a stabilization energy in the range of ca. 17 and 23 kcal mol^−1^. In both cases, the simulated PXRD pattern resulting from the stabilized structures (Figure 1A, C blue lines, Figure S3) reveals a better matching with the experimental pattern, now including the broad peaks at 2θ≈13.0–14.0° and at 2θ≈26.0–27.0°. This observation indicates that the structural arrangement of both COFs consists of slip‐stacked layers with a random inter‐layer offset.^[23]^ The PXRD broad features indicative of a COF interlayer slipped arrangement were expected when using asymmetric building blocks, as reported by Lotsch et al.[^24^, ^25^]

These structural features are further confirmed by N_2_ adsorption experiments. Both COFs exhibit Type IV isotherms typical of mesoporous materials (Figure S4), with initial adsorption into the pores at low relative pressures (0.01<P/P_0_<0.10).^[26]^ The Brunauer–Emmett–Teller (BET) surface‐area model was applied providing BET surface areas of 472 m^2^ g^−1^ and 691 m^2^ g^−1^ for **COF‐IsoQ‐Tp** and **COF‐Naph‐Tp** respectively. Application of non‐local density functional theory (NLDFT) models over the measured isotherms yielded a similar pore size distribution for both COFs centered at 1.1 nm closely matching the theoretical pore size of 1.4 nm, albeit spanning in the range 0.6–2.0 nm in reasonable agreement with the slip‐stacked modeled structure (Figure S2).^[27]^

X‐ray photoelectron spectroscopy (XPS, Figures S5–6) provides direct evidence of the **COF‐Naph‐Tp** and **COF‐IsoQ‐Tp** structural analogy while highlighting the N‐doping diversity. Indeed, the C 1s spectra of the two materials confirm the formation of the β‐ketoenamine backbone as in both cases (**COF‐IsoQ‐Tp**, vs **COF‐Naph‐Tp**) the signal can be deconvoluted into three peaks, assigned to sp^2^ carbon (284.3 eV 55 % vs 284.3 eV 66 %), C−N carbon (285.6 eV 38 % vs 285.5 eV 29 %) and C=O carbon (287.8 eV 7 % vs 287.5 eV 5 %).^[28]^ As expected, the N 1s spectrum of **COF‐Naph‐Tp** shows one peak centered at 398.9 eV which is ascribed to the enamine nitrogen (−C=C−NH), while for **COF‐IsoQ‐Tp**, deconvolution of the N 1s signal results into two peaks, one corresponding to the enaminic nitrogen at 398.8 eV (59 %) and the other centered at 397.7 eV (41 %) attributed to the pyridinic nitrogen species,^[29]^ this latter resulting from COF incorporation of the isoquinoline component.

Thermogravimetric analysis (TGA, Figure S8) performed under inert atmosphere, indicates that both COFs undergo a gradual weight loss (ca 40 %) above 400 °C, likely corresponding to the framework decomposition, while showing no evidence of volatile guest adsorbates.

**Figure 2:**
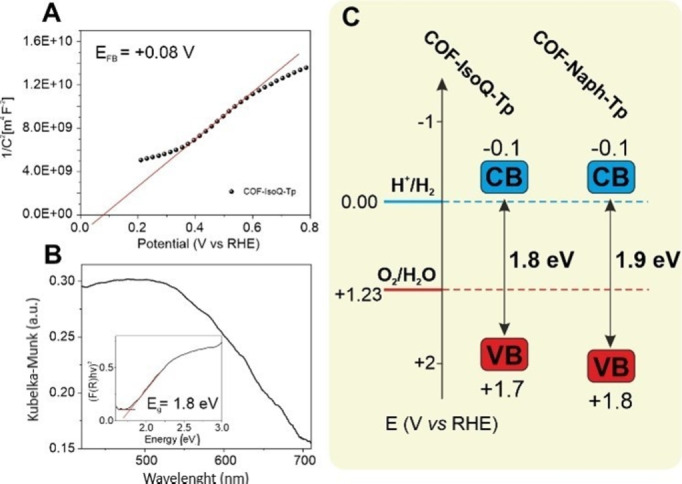
(A) Mott‐Schottky plot of **COF‐IsoQ‐Tp** electrode at 1000 Hz (0.1 M aqueous Na_2_SO_4_, pH=7). (B) Representative diffuse reflectance spectrum of **COF‐IsoQ‐Tp** and corresponding Tauc plot (inset). (C) Band gap and band position of **COF‐IsoQ‐Tp** and **COF‐Naph‐Tp** against reversible hydrogen electrode (RHE) along with the potential of H^+^/H_2_ and O_2_/H_2_O.

The UV‐vis diffuse reflectance (DR, Figure S9) spectra of **COF‐Naph‐Tp** and **COF‐IsoQ‐Tp** are characterized by an extended light absorption up to ca. 700 nm, when compared to the p‐phenylenediamine (Pa) analog, TpPa‐COF, featuring an absorption band edge at ca. 615 nm.^[21]^ This light‐harvesting capacity is therefore ascribed to an improved π conjugation across the COF network imparted by the naphthalene and isoquinoline rings. Both COFs share similar optical properties as the Kubelka‐Munk′s analysis of their DR spectra yields comparable optical band gaps (1.9 eV and 1.8 eV for **COF‐Naph‐Tp** and **COF‐IsoQ‐Tp** respectively, Figures 2 and S9). Similar photoluminescence spectra (PL, λ_exc_=420 nm, Figure S10) are obtained with emission maxima at ca. λ_max_=460 nm and ca. λ_max_=487 nm. These PL envelopes can be correlated to the emission of the conjugated β‐ketoenamine residues present in the polymerized frameworks.[^30^, ^31^] Moreover, both COFs show similar PL dynamics, with lifetimes of 1.15±0.01 and 1.23±0.01 ns determined for **COF‐IsoQ‐Tp** and **COF‐Naph‐Tp** respectively (Figure S11, Table S3).

Mott‐Schottky (MS) plots (Figure S12) of COF‐modified FTO working electrodes (COF@FTO, Figures S13–17, Table S4) indicate that both COFs behave as *n*‐type semiconducting materials. This is also confirmed by plane‐wave DFT calculations where the Fermi level is located close to the conduction band (E_CB_). The flat‐band potential (E_FB_) is derived from the x‐intercept of the linear region in the Mott‐Schottky plots, and its values are determined to be 0.09 V and 0.08 V vs RHE for **COF‐Naph‐Tp** and **COF‐IsoQ−Tp**, respectively (Figure S12). This places the conduction band for both COFs at approximately E_CB_=−0.1 V, considering that for n‐type semiconductors, the E_CB_ is slightly more negative, approximately ca. −0.2 V, than the corresponding flat‐band potential.^[32]^ Thus, both COFs should possess sufficient thermodynamic driving force for proton reduction to hydrogen (E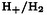 =0.00 V *vs* RHE) although with a narrow margin (~100 mV) (Figure 2). In addition, the valence band (E_VB_) is determined to be 1.8 and 1.7 V *vs* RHE for **COF‐Naph‐Tp** and **COF‐IsoQ‐Tp** respectively, thus possessing a sufficient oxidation potential for reaction with sacrificial electron donors like ascorbic acid (AA), reaching out to the most demanding oxygen evolution reaction (E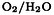 =+1.23 V *vs* RHE).

The COF@FTO photocurrent behavior was addressed by photoelectrochemical experiments under light‐chopped illumination in a three‐electrode cell configuration, (solar simulator AM 1.5 G 200 mW cm^−2^, nitrogen‐purged 0.1 M aqueous Na_2_SO_4_). Both COF‐based photoelectrodes showed cathodic photocurrent with similar onset (ca. +0.8 V *vs* RHE), however reaching remarkably different values, whereby **COF‐IsoQ‐Tp** outperforms **COF‐Naph‐Tp** by a 300 % photocurrent enhancement (respectively J=−12±3 μA cm^−2^ and −4±2 μA cm^−2^ at 0.68 V *vs* RHE, Figures S18 and 19, Tables S5 and 6). Notably, the isoquinoline‐based material outperforms most COF‐based photoelectrodes reported in the literature so far (Table S7), featuring a photocurrent loss as low as 20 % after 300 s under chopped chronoamperometry (CA) conditions.

Photoelectrochemical impedance spectroscopy (PEIS) experiments were instrumental to compare and contrast the photoconductivity properties of **COF‐IsoQ‐Tp** and **COF‐Naph‐Tp**.

In particular, Nyquist plots analysis and data fitting, via a modified Randles circuit, indicate the interplay of two photo‐induced charge‐transfer resistance terms (Figures S20 and 21), for both COF‐based photoelectrodes, namely: (i) a bulk charge transport term (R_t_=R_1_ in Figure S20) and (ii) a solid‐liquid interfacial resistance, ascribed to the charge transfer to the aqueous electrolyte phase (R_ct_=R_2_ in Figure S20). PEIS results show a remarkable enhancement of the photoconductivity capacity for the isoquinoline‐based COF, with bulk resistance decreased by ca. one order of magnitude compared to the naphtalene analog (R_t_=3±1 KΩ, and 21±2 KΩ respectively). Notably, R_t_ values lower than 3 KΩ, are found only when COF materials are built as a heterojunction with inorganic semiconductors (e.g. TiO_2_, details in Table S8).^[33]^ These results suggest that the COF N_*Pyridine*_‐carbon doping, significantly contributes to improve the charge carrier transport,[^34^, ^35^] while having a negligible impact on the charge transfer properties of the interfacial water under operando conditions.

Indeed, the similar values of the R_ct_ term obtained for both photoelectrodes (R_ct_=16±4 KΩ and 10±4 KΩ for **COF‐IsoQ‐Tp** and **COF‐Naph‐Tp** respectively) indicate a similar interfacial interaction with the aqueous phase under photoelectrochemical operando conditions.[^36^, ^37^]

**Figure 3:**
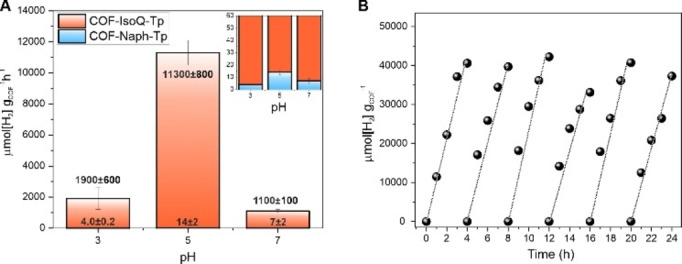
(A) COF‐mediated photocatalytic HER as a function of pH in presence of 4 % platinum nominal loading. Inset: zoom‐in to visualize the **COF‐Naph‐Tp** activity. (B) Recyclability test of **COF‐IsoQ‐Tp** in photocatalytic HER (see conditions of entry iv in Table 1).

**COF‐mediated Photocatalytic Hydrogen Evolution Reaction (PHE)**. The photocatalytic performance of both COFs was assessed for light‐assisted H_2_ evolution with Pt nanoparticles (PtNPs) as co‐catalysts and in the presence of ascorbic acid (AA) as the sacrificial electron donor (SED). In a standard photocatalytic experiment, 2.5 mg of COF powder were dispersed via ultrasonication in a 5 mL aqueous buffer (Britton‐Robinson Buffer, BRB) solution, along with AA (75 mM) and hexachloroplatinic acid as catalyst precursor. The photocatalytic ensemble was then irradiated using a solar simulator (λ>400 nm, AM 1.5 G, 6 suns illumination), while the hydrogen evolution rate was evaluated as a function of both pH and Pt loading conditions (Table 1, Figure S22). Across all examined conditions, **COF‐IsoQ‐Tp** turned out to outperform **COF‐Naph‐Tp**, with HER peaking at 11300±800 μmol g^−1^ h^−1^ under pH 5 and 4 % PtNPs loading conditions (entry iv, Table 1), thus showcasing a PHE rate with three‐order magnitude enhancement compared to **COF‐Naph‐Tp** (entry vii, Table 1 and Figure 3A). In particular, both COFs feature a bell‐shaped HER trend as a function of pH, with the top performance observed at pH 5 (Figure 3A), however with a diverse pH‐modulation effect, as a tenfold rate increase is demonstrated for **COF‐IsoQ‐Tp** when moving from pH 7 to pH 5 (entries iii and iv, Table 1), while photocatalysis by **COF‐Naph‐Tp** remains negligible under the same conditions (entries vi and vii, Table 1). The observed bell‐shaped PHE‐pH profile points to a concurrent interplay of factors that regulate photocatalysis, namely: favored proton transfer and metal‐hydride formation at lower pH, counterbalanced by the light‐activated reducing quenching involving AA (p*K*_a_=4.1) which is promoted at higher pH, due to a more favorable redox potential of the ascorbate anion. Indeed, similar bell‐shaped profiles have been reported for PHE using AA as sacrificial electron donor.[^38^, ^39^, ^40^, ^41^]

**Table 1:** Comparison of PHE figures of merit for **COF‐IsoQ‐Tp** and **COF‐Naph‐Tp** as function of pH and Pt loading conditions.

Entry	System	pH^[a]^	%Pt^[b]^	PHE^[c]^ μ mol g^−1^ h^−1^]
(i)	**COF‐IsoQ‐Tp**	7	0.1	600±100
(ii)	**COF‐IsoQ‐Tp**	7	1	3300±500
(iii)	**COF‐IsoQ‐Tp**	7	4	1100±100
(iv)	**COF‐IsoQ‐Tp**	5	4	11300±800
(v)	**COF‐IsoQ‐Tp**	3	4	1900±600
(vi)	**COF‐Naph‐Tp**	7	4	7±2
(vii)	**COF‐Naph‐Tp**	5	4	14±2
(viii)	**COF‐Naph‐Tp**	3	4	4.0±0.2

In all experiments: 2.5 mg of COF are dispersed in a 5 mL aqueous buffer (Britton‐Robinson Buffer, BRB 0.5 M) solution, with AA (75 mM) and hexachloroplatinic acid (0.8 % w/w) under AM 1.5 G, 6 suns illumination, (cut‐off filter λ >400 nm). [a] pH corrected by adding concentrated HCl and NaOH solution to BRB. [b] w/w% calculated on the nominal Pt precursor versus the amount of COF. [c] Rate of hydrogen evolution calculated by linear kinetic fitting of 3 experiments.

Noteworthy, no loss of photocatalytic stability was observed for **COF‐IsoQ‐Tp** during a 24 h long experiment under optimized conditions (entry iv, Table 1, Figures S23–24), in which the reactor headspace was purged every 4 h and the sacrificial electron donor was refilled every 8 h (Figure 3B). Under the conditions explored, the **COF‐IsoQ‐Tp** works with an apparent quantum yield, AQY= 0.2 % at λ=456 nm (SI, Section 1.2), which is similar to what is obtained with other **Tp**‐based COFs.^[20]^ The low PHE activity of **COF‐Naph‐Tp** prevents an accurate determination of the AQY value.

In order to address the orders of magnitude, ON‐OFF switching of PHE observed for **COF‐IsoQ‐Tp** and **COF‐Naph‐Tp**, additional evidence on the Pt‐active clusters have been collected for both COFs after photodeposition of PtNPs. Inspection of transmission electron microscopy (TEM) images shows a uniform dispersion of PtNPs with an average nanoparticle size of ca. 2–3 nm, generated under photocatalytic conditions from the Pt molecular precursor respectively at pH 7 and 5 on both COFs, (Figures S25 and 26). The PtNPs size closely resembles the typical NP dimensions obtained upon photodeposition mediated by COFs according to literature protocols (Table S1). As a result, the platinum loading in both COF systems, as determined by ICP‐OES, turns out to be comparable (Tables S9–12). Therefore, the specific activation of the PtNPs on the **COF‐IsoQ‐Tp** has been further investigated by XPS analysis.

**Figure 4:**
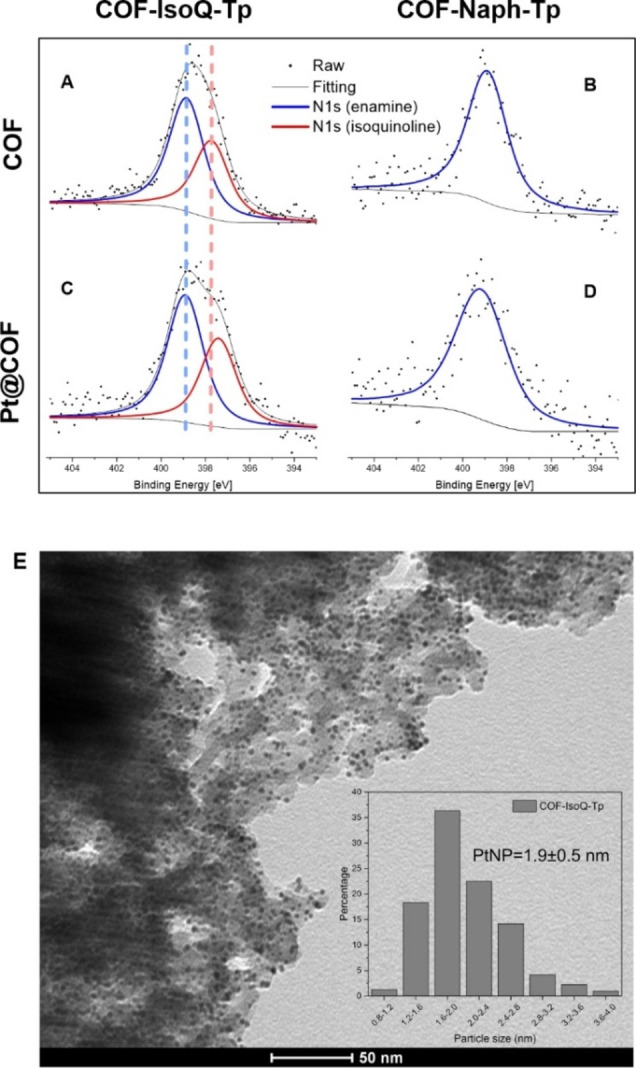
N 1s XPS region of (A) **COF‐IsoQ‐Tp**, (B) **COF‐Naph‐Tp** (C) **Pt@COF‐IsoQ−Tp**, and (D) **Pt@COF‐Naph‐Tp**. (E) TEM image of **COF‐IsoQ‐Tp** showing the PtNPs formation. The inset displays the distribution of PtNP sizes.

In particular, XPS characterization was performed to study the surface and local structures of **Pt@COF‐IsoQ‐Tp** over **Pt@COF‐Naph‐Tp** by comparison with the pristine COFs. The N 1s signal of both Pt‐loaded COF, displays a minor positive peak shift of 0.2 eV, which is consistent with a weak interaction between the Pt clusters and the β‐ketoenamine nitrogen for both materials (Figure 4B and D). On the contrary, **Pt@COF‐IsoQ‐Tp** shows a unique negative peak shift of 0.7 eV that is emerging from a specific interaction of Pt‐clusters with the pyridinic nitrogen in **COF‐IsoQ‐Tp**. This downshifted binding energy of N 1s emerges upon formation of a Pt^0^‐N bond where the pyridinic N in **Pt@COF‐IsoQ‐Tp** is electron‐rich (Figure 4A and C).[^42^, ^43^] Specifically, the peak at 399.1 eV, present in both Pt@COF, is ascribed to the enamine nitrogen (−C=C−NH), while the peak at 397.0 eV in the N 1s XPS spectrum of **Pt@COF‐IsoQ‐Tp** is indexed as Pt‐N_Pyridine_‐C (Figure 4C). For Pt 4 f XPS spectra (Figure S7), as expected, both **Pt@COF‐IsoQ‐Tp** and **Pt@COF‐Naph‐Tp** consisted of Pt(0). Combining the results from N 1s XPS spectra, we concluded that the interaction between Pt NPs and COF in **Pt@COF‐IsoQ‐Tp** led to the electron transfer from Pt to N. These results confirm the formation of extended Pt‐N_Pyridine_‐C heterojunction at the isoquinoline domains in **Pt@COF‐IsoQ‐Tp**, which are likely responsible for boosting PHE.

DFT calculations corroborate the XPS experimental results by modelling a Pt_38_ cluster interacting with a **COF‐IsoQ‐Tp** pore (Figure S31). It turns out that the electron density of the isoquinoline nitrogen increases upon Pt^0^‐N bonding, while a reverse effect occurs at the Pt atom. This is supposed to play a crucial role for photoinduced electron and proton transfer at the COF reactive sites.^[44]^ This electronic interaction mediated by the isoquinoline moiety, constitutes a functional Schottky heterojunction within the COF, facilitating the electron collection at the PtNP co‐catalyst under photocatalytic regime.^[45]^ Noteworthy, DFT calculations point to an overall increase of electron density on the Pt_38_ cluster, as expected upon formation of a metal‐semiconductor heterojunction.^[46]^

Therefore, both structural and spectroscopic evidence suggest a reaction‐site model where the active PtNPs are co‐localized at the COF isoquinoline domains, establishing functional Schottky heterojunctions between the isoquinoline nitrogen atoms and the Pt clusters, leading to efficient photocatalytic hydrogen evolution (Figure 1).

## Conclusions

Our results highlight the key enabling potential of materials design by a bottom‐up molecular synthesis that is strategic for functional frameworks as COFs. Herein, ordered arrays of N‐doped photocatalytic sites are placed into action within a porous β‐ketoenamine carbon framework by reticulating 5,8‐diaminoisoquinoline (**IsoQ**) with triformylphloroglucinol (**Tp**) precursors into **COF‐IsoQ‐Tp**. In this asset and compared to the isostructural isoquinoline‐free analog (**COF‐Naph‐Tp)**, the N‐rich heterocycle domains turn out to leverage photocatalytic hydrogen evolution by co‐localized PtNPs, with three orders of magnitude higher rates (11300 μmol g^−1^ h^−1^ vs 14 μmol g^−1^ h^−1^). Converging photoelectrochemical impedance spectroscopy (PEIS), XPS and DFT evidence indicate that PtNP bonding occurs at the N‐rich COF pores thus triggering multiple Schottky heterojunctions that facilitate proton and electron transfer, charge separation and transport between the photoactive COF and the metal cores. COF materials embedding N‐based coordinating cores including heteroaromatic bases, can provide a unique platform to dissect structure‐reactivity relationships to address precision chemistry for energy research.

## Conflict of Interests

The authors declare no conflict of interest.

1

## Supporting information

As a service to our authors and readers, this journal provides supporting information supplied by the authors. Such materials are peer reviewed and may be re‐organized for online delivery, but are not copy‐edited or typeset. Technical support issues arising from supporting information (other than missing files) should be addressed to the authors.

Supporting Information

## Data Availability

The data that support the findings of this study are available in the Supporting Information of this article.
