# Supplementary material for: ON‐OFF Switching of Photocatalytic Hydrogen Evolution by Built‐in Pt‐Nitrogen‐Carbon Reticular Heterojunctions
Source: ChemSusChem. 2024 Nov 14;18(5):e202401977. doi: 10.1002/cssc.202401977 (PMC11874682; doi:10.1002/cssc.202401977)
Supplement: Supplementary file 1 — Supporting Information [file CSSC-18-e202401977-s001.pdf]

ChemSusChem

Supporting Information

ON-OFF Switching of Photocatalytic Hydrogen Evolution by Built-in Pt-Nitrogen-Carbon Reticular Heterojunctions

Leonardo Cognigni, Thomas Gobbato, Elisabetta Benazzi, Lorenzo Paoloni, Biagio Di Vizio, Ruggero Bonetto, Francesco Rigodanza, Alessandro Bonetto, Stefano Agnoli, Marcella Bonchio,* and Paolo Costa*

ON-OFF switching of Photocatalytic Hydrogen Evolution by Built-in Pt-Nitrogen-Carbon Reticular Heterojunctions

Leonardo Cognigni, Thomas Gobbato, Elisabetta Benazzi, Lorenzo Paoloni, Biagio Di Vizio, Ruggero Bonetto, Francesco Rigodanza, Alessandro Bonetto, Stefano Agnoli, Marcella Bonchio,* Paolo Costa*

S1: Materials, Instrumentations, and Method 3
S1.1: Materials and Instrumentations 3
S1.2: Photocatalytic hydrogen evolution reaction (HER) experiments 4
S1.3: Electrochemical and Photoelectrochemical experiments 4
S2: Synthetic Procedure 5
S2.1: 2,4,6-Triformylphloroglucinol (Tp) Synthesis 5
S2.2: 5,8-diaminoisoquinoline Synthesis 5
S2.3: 1H-NMR and 13C-NMR Spectra 6
S2.4: Covalent Organic Frameworks Synthesis 9
S2.5: Electrodes Fabrication 9
S3: Supporting Figures 10
S3.1: Benchmarking of state-of-the-art COF photocatalysts HER activity 10
S3.2: FT-IR Analysis 11
S3.3: Elemental Analysis 11
S3.4: PXRD Analysis 12
S3.5: BET Analysis 14
S3.6: X-Ray Photoelectron Spectroscopy (XPS) 15
S3.7: Thermogravimetric Analysis (TGA) 16
S3.8: Diffuse Reflectance 17
S3.9: Photoluminescence 18
S3.10: Mott-Schottky Spectroscopy 19
S3.11: Electrode Characterization 19
S3.12: Photoelectrochemical Experiments 22
S3.13: Photocatalytic Hydrogen Evolution 27
S3.14: Transmission Electron Microscopy (TEM) 29
S3.15: Pt Loading by ICP-OES 31
S4: Theoretical Calculations 32
S4.1 COF-IsoQ-Tp and COF-Naph-Tp structural and optoelectronic properties 32
S4.2 COF-IsoQ-Tp/Pt_{38} cluster interaction 35
S5: References 36

S1: Materials, Instrumentations, and Method

S1.1: Materials and Instrumentations

Chemicals and solvents for all synthetic and characterization procedures have been purchased from SigmaAldrich and Fluorochem and used as received, if not specified. Deuterated solvents have been purchased from Sigma-Aldrich. Alconox and TEC 8 Fluorine-doped Tin Oxide (FTO) conductive glass slides were obtained from Sigma Aldrich.
NMR spectra were recorded on Bruker 300 equipped with a BBI-ATM-z grad probe head 5 mm. The chemical shifts (δ) for 1H and 13C are given in ppm relative to residual signals of the solvents ($CHCl_3$ @ 7.26 ppm 1H NMR, 77.16 ppm 13C NMR; CD_3CN @ 2.13 ppm and 1.94 ppm ^{1}H NMR, 118.26 ppm and 1.32 ppm ^{13}C NMR). Coupling constants are given in Hz. The following abbreviations are used to indicate the multiplicity: s, singlet; d, doublet; t, triplet; q, quartet; m, multiplet; bs, broad signal.
Powder XRD (PXRD) measurements have been performed on a Bruker D8 diffractometer equipped with a Göbel mirror and a CuKα X-ray source.
Fourier-Transform Infrared (FT-IR) spectra have been recorded on a Varian 660 FT-IR with KBr pellets.
Spectral reflectance measurements were recorded with an integrating sphere (120 mm in diameter and BenFlect inner surface coating) fully integrated in the sample compartment of the Edinburgh photoluminescence spectrometer model FLS1000, equipped with double monochromators, a 450 W Xe arc lamp as the excitation source and an extended red photomultiplier (Hamamatsu R13456, spectral response 185 – 980 nm) as the detector. For the reflectance spectra, the step and dwell time were set at 1 nm and 0.2 s, respectively, and the slit was kept at 2 nm and 0.2 nm for excitation and emission monochromators, respectively. Reflectance was expressed in Kubelka-Munk units, according to the equation:

$$\frac{k}{s} = \frac{(1 - R_{\propto})^2}{2R_{\propto}}$$

Fluorescence decay dynamics studies have been performed using 405 nm laser pulses on a FLS1000 by Edinburgh Instruments equipped with a PMT-980 detector.
SEM was performed with a Zeiss Sigma HD microscope, equipped with a Schottky FEG source, one detector for backscattered electrons, and two detectors for secondary electrons (InLens and Everhart Thornley). The microscope is coupled to an EDX detector (from Oxford Instruments, x-act PentaFET Precision) for X-rays microanalysis, working in energy dispersive mode.
X-Ray Photoelectron Spectroscopy: The surface chemical characterization of the materials has been carried out using XPS in a custom-made UHV system working at a base pressure of 10^{-10} mbar, equipped with an Omicron EA125 electron analyzer and an Omicron DAR 400 X-ray source with a dual Al−Mg anode. Core-level photoemission spectra (C 1s, N 1s, and Pt 4f) were collected at rt with a non-monochromatic Al Kα X-ray source (1486.3 eV) and using an energy step of 0.1 eV, 0.5 s of integration time, and 20 eV of pass energy. The samples were suspended in methanol and drop cast on Cu metal or glassy carbon supports. For Pt-loaded samples, the electron charging was neutralized by a flood gun. The binding energy scale was referenced to the carbon C-C bond in the C1s photoemission line at 284.8 eV.
TEM analysis was performed with a FEI Tecnai G2 12 transmission electron microscope operating at 100 kV and equipped with a TVIPS CCD camera.
Thermogravimetric analyses were carried out with Q5000IR TGA (TA Instruments) under nitrogen by an isotherm at 50°C for 15 minutes followed by heating at 10°C/min until 800°C
BET: The textural properties of the samples were studied by N_2 adsorption at 77 K with a static volumetric instrument (ASAP2020, Micrometrics) over a wide relative pressure range from about 10^{-6} to 0.998 (p/p_0). Prior to the analysis, all the samples were degassed at 383 K for 14 h under vacuum (about $1x10^{-1}$ Pa) to completely remove any trace of adsorbed water. The specific surface area of the samples was derived by applying the standard Brunauer–Emmett–Teller (BET) method, using N_2 adsorption data in the relative pressure range indicated by Rouquerol parameter.[1] Non-Local Density Functional Theory (NL-DFT) was also used to fit experimental data exploiting the MicroActive software provided by Micrometrics, according to the approach of Jagiello,[2] namely HS-2D-NLDFT model, thus pore size distribution, covering the full range of micro and mesopores width, was derived.
ICP-OES: Around 5 mg of sample has been weighted (Practum, Sartorius, 0.01mg) and inserted in Teflon vessel for the acidic mineralization assisted by microwave (Ethos UP, Milestone). A mixture of 5 ml of Acqua Regia (3:1 $HCl:HNO_3$) and 2 ml of ultrapure H_2O_2 has been added to the vessel, and the container has been sealed and microwave heated following the ramp reported in Table A. The solutions are then diluted with a final volume of 50 ml with ultrapure water and diluted again 10 times for the analysis. Samples has been analysed with ICP-MS Perkin Elmer Nexion 350XX using Rh 103 as internal standard. Pt 195 has been quantified by means an external six-point calibration curves from 20 to 200 µg/L in KED mode, using He as collision gas (4.4. mL).

Table A. Ethos UP's temperature Ramp

Time	Temperature (°C)
20	210
15	210
Cooling	20 min
Program Name	Activated Carbon 90%

S1.2: Photocatalytic hydrogen evolution reaction (HER) experiments

All photocatalysis experiments were performed in a Schlenk tube (Ø= 1 cm), and fans were employed for cooling the reactor. The reactor was irradiated with a calibrated LOT-QuantumDesign solar simulator, equipped with an AM 1.5 G filter plus a 400 nm cut-off filter (600 mW cm^{-2}). For each experiment, the photocatalyst (COF; 2.5 mg) was suspended in Britton-Robinson Buffer (5 mL of 0.5 M solutions at pH 3-7) containing Ascorbic acid as sacrificial electron donor (65 mg; 0.370 mmol). Hexachloroplatinic acid (25 µL, 0.8 wt% aqueous solution, SigmaAldrich) was added for the in-situ formation of platinum nanoparticles as the co-catalyst. Before the irradiation, the reactor was purged with nitrogen for 15 m minutes.
In the course of the experiment, the headspace of the reactor was periodically sampled, and the components were quantified by gas chromatography (Agilent Technologies 7890A GC System) equipped with a thermal conductivity detector (TCD) detector using argon as the carrier gas.
For long-term photocatalysis experiments, the headspace of the reactor was evacuated and purged with argon every 4 hours to avoid hydrogen buildup and the photocatalysis was resumed. Ascorbic acid was regenerated every 8 hours.
Apparent Quantum Yields (AQY) experiments. were determined under irradiation using Kessil lamp filters centered at 456 nm (60 mW cm^{-2}). For this purpose, 2.5 mg of covalent organic framework (COF) was suspended in buffer (BRB, 5 mL of 0.5 M solution at pH 5) containing Ascorbic acid (65 mg 0.7 mmol) and hexachloroplatinic acid (25 µL, 0.8 wt% aqueous solution,Sigma-Aldrich). The AQY was then calculated using the equation $AQY=2\cdot[H_2]/I$, where $[H_2]$ is the average hydrogen evolution rate and I is the incident photon flux, calculated through a ferrioxalate actinometry protocol. [3]

S1.3: Electrochemical and Photoelectrochemical experiments

Electrochemical and photoelectrochemical experiments were carried out in sodium sulphate Na_2SO_4 0.1 M, pH 7, on a PGSTAT302N potentiostat in a three-electrode configuration using as working electrode COF-IsoQ-Tp@FTO or COF-Naph-Tp@FTO electrodes, as counter electrode Au and as reference electrode Ag/AgCl (NaCl 3 M, BASi). Potentials are then converted to RHE using the correlation $V(\text{RHE}) = V(Ag/AgCl) + 0.205\text{ V} + 0.059\text{ V}\cdot\text{pH}$ A LOT-QuantumDesign solar simulator, equipped with an AM 1.5 G filter, was used as the illumination source. The power of the light source for light management experiments was measured using a THOR LABS PM100D power meter coupled with a CCD THOR LABS S370C, back illumination was adopted for all the experiments and unless otherwise stated, the majority of the measurements were recorded at 200 mW cm^{-2}. Current-voltage curves were all recorded at a scan rate of 10 mV s^{-1}. Current-voltage and current-time curves under chopped illumination were acquired by manually chopping the excitation source.
Photoelectrochemical Impedance Spectroscopy (PEIS) experiments were acquired with an Autolab PGSTAT302N potentiostat coupled with a FRA32M module and Nova electrochemical software. PEIS data were registered at an applied potential of 0.4 *vs* RHE using the experimental setup of photoelectrochemical experiments and by applying a 10 mV amplitude perturbation with frequencies between 10^5 and 0.1 Hz.
Mott-Schottky measurements were conducted in dark using the experimental setup of photoelectrochemical experiments. M-S plots were obtained applying 40 equidistant bias potentials from 0.2 to 0.9 *vs* RHE (1000 Hz applied frequency, 0.01 amplitude)

S2: Synthetic Procedure

S2.1: 2,4,6-Triformylphloroglucinol (Tp) Synthesis

Adapted from previously reported procedures. [4]

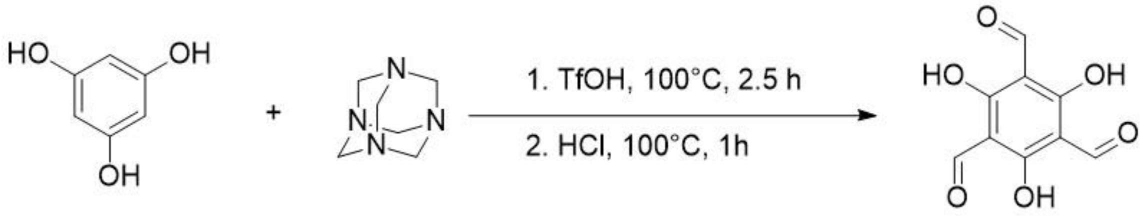

Scheme S1. Synthetic scheme of 2,4,6-Triformylphloroglucinol.

To hexamethylenetetramine (15.098 g, 108 mmol) and dried phloroglucinol (6.014 g, 49 mmol) trifluoroacetic acid was added 90 mL under N_2 atmosphere. The solution was heated at 100 ºC for 2.5 h. Approximately 150 mL of 3 M HCl was added, and the solution was heated at 100 ºC for 1 h. After cooling to room temperature, the solution was filtered, extracted with ca. 350 mL dichloromethane, dried over magnesium sulfate, and filtered. Rotary evaporation of the solution afforded 1.15 g (5.5 mmol, 11%) of an off-white powder.
1H NMR (300 MHz, $CDCl_3$) δ 14.12 (s, 3H), 10.15 (s, 3H) ppm; ^{13}C-NMR (101 MHz, $CDCl_3$) δ 192.21, 173.72, 103.03.

S2.2: 5,8-diaminoisoquinoline Synthesis

Adapted from previously reported procedures. [5,6]

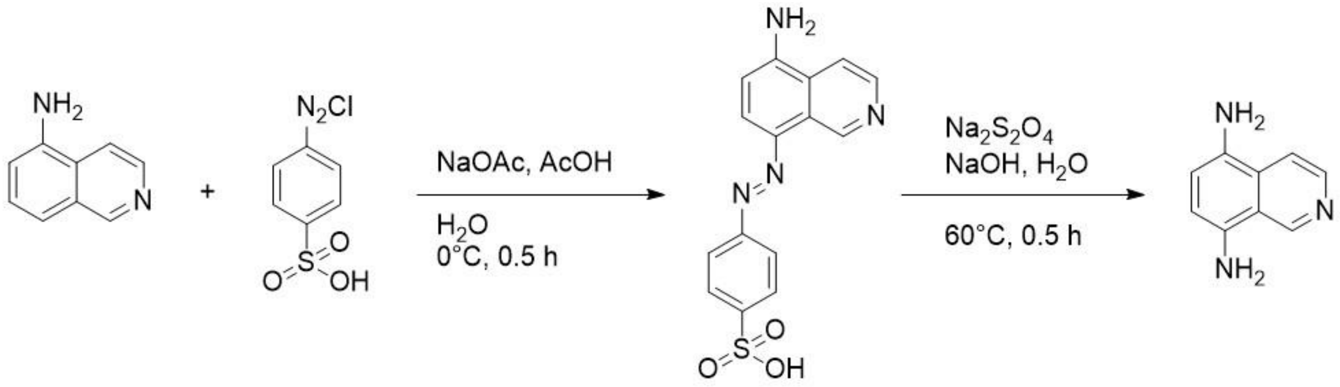

Scheme S2. Synthetic scheme of 5,8-diaminoisoquinoline.

5-Aminoisoquinoline (1.44 g., 0.01 mol) was dissolved in a mixture of 100 ml. of 1 N acetic acid and 100 ml. of a saturated sodium acetate solution. A slurry of diazotized sulfanilic acid [7] (2.44 g., 0.014 mol) was added to this solution at 0°, with stirring. After 30 min., the product was salted out with NaCl. The moist azo dye was suspended in 60 ml. of water containing 1.6 g. of sodium hydroxide, heated to 50°, and treated with solid sodium hydrosulfite (30 g.). The red solution turned brown, and the temperature spontaneously rose to 60 °C. It was kept at 60 °C for 30 min and finally cooled to room temperature. The solution was made strongly alkaline with sodium hydroxide (5.0 g) and was rapidly extracted with DCM (7 X 200 mL). The combined organic solution was washed with saturated sodium chloride (2 X 100 mL), dried (Na_2SO_4), filtered, and evaporated to dryness. The diamino compound was obtained by recrystallization from hot acetonitrile as a brown crystalline solid, affording 0.9 g (0.006 mol, 60%).
1H NMR (300 MHz, $CDCl_3$): δ [ppm] 9.31 (s,1H), 8.51 (d, 5.94 Hz,1H), 7.59 (d, 5.95 Hz,1H), 6.86 (d, 7.81 Hz,1H), 6.72 (d, 7.84 Hz, 1H), 4.40-3.30 (br, 4H)
13C NMR (100.6 MHz, CD_3CN) δ [ppm] 148.34, 142.57, 137.24, 135.02, 127.59, 120.40, 115.79, 115.58, 112.51
HRMS (ESI): Calcd for $C_9H_{10}N_3$ $[M+H]^+$: 160.0869, found 160.0888

S2.3: 1H-NMR and 13C-NMR Spectra

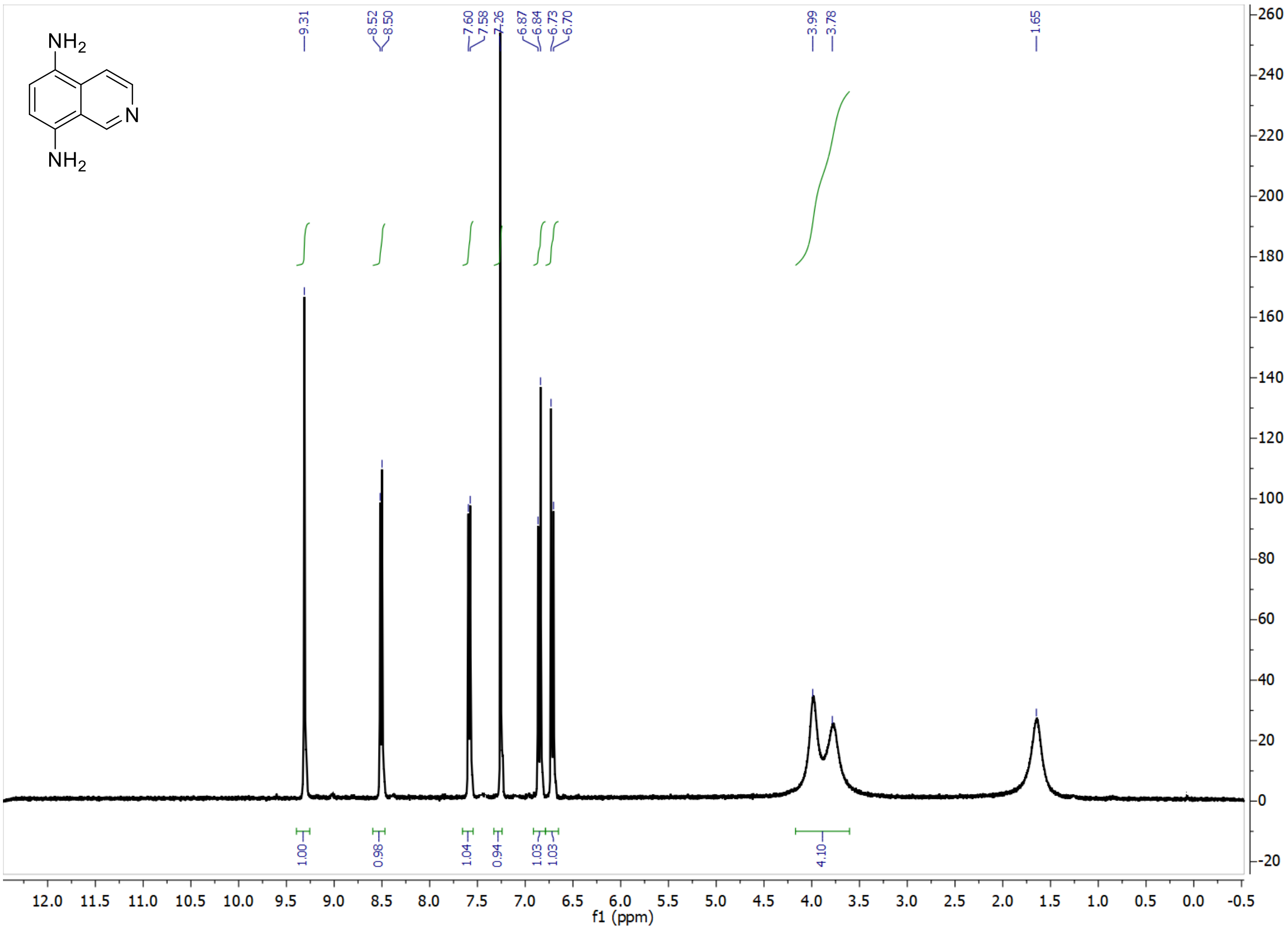

^{1}H-NMR (300 MHz, $CDCl_3$) of 5,8-diaminoisoquinoline.

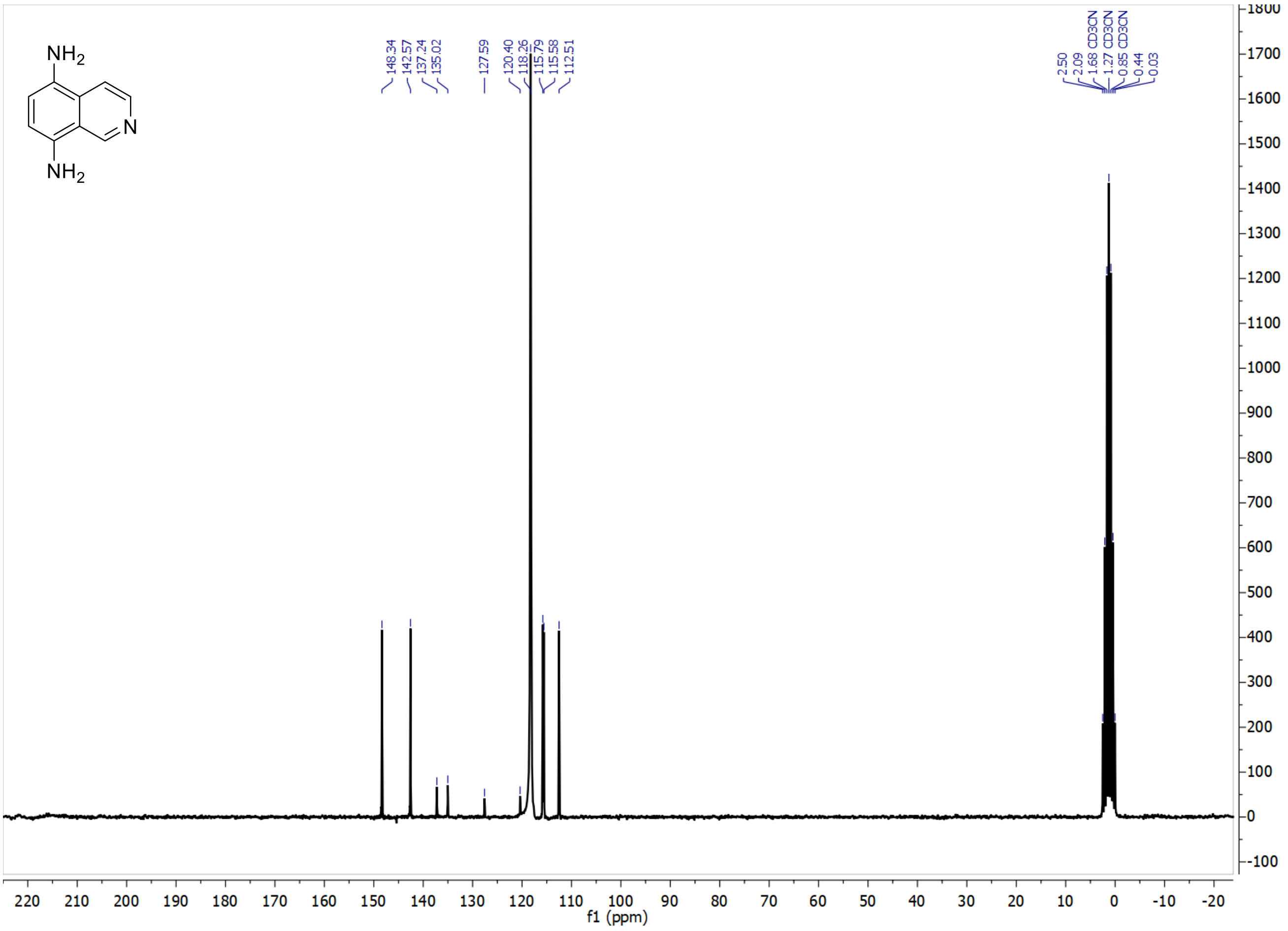

^{13}C-NMR (100.6 MHz, CD_3CN) of 5,8-diaminoisoquinoline.

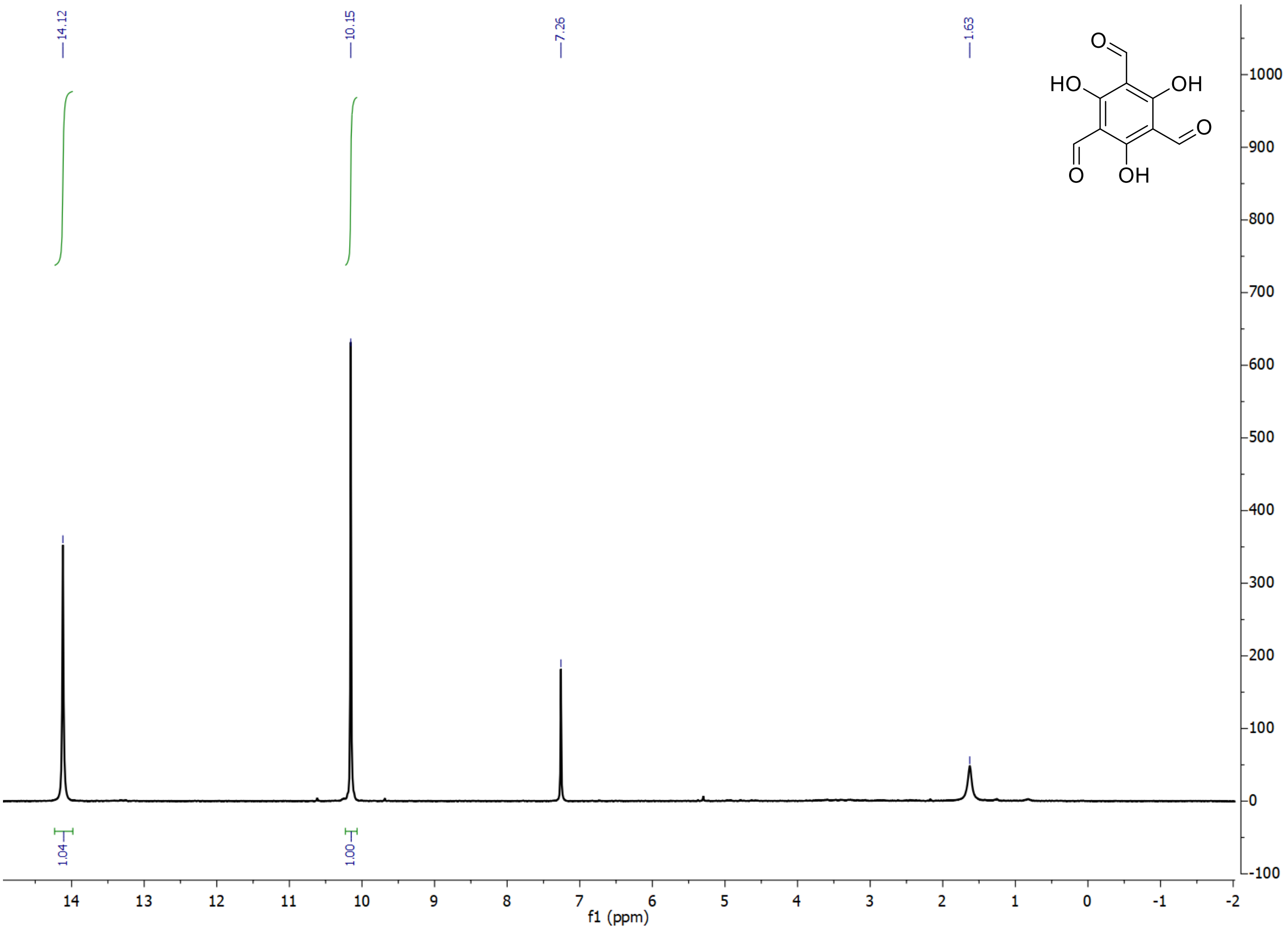

^{1}H-NMR (300 MHz, $CDCl_3$) of 2,4,6-Triformylphloroglucinol.

S2.4: Covalent Organic Frameworks Synthesis

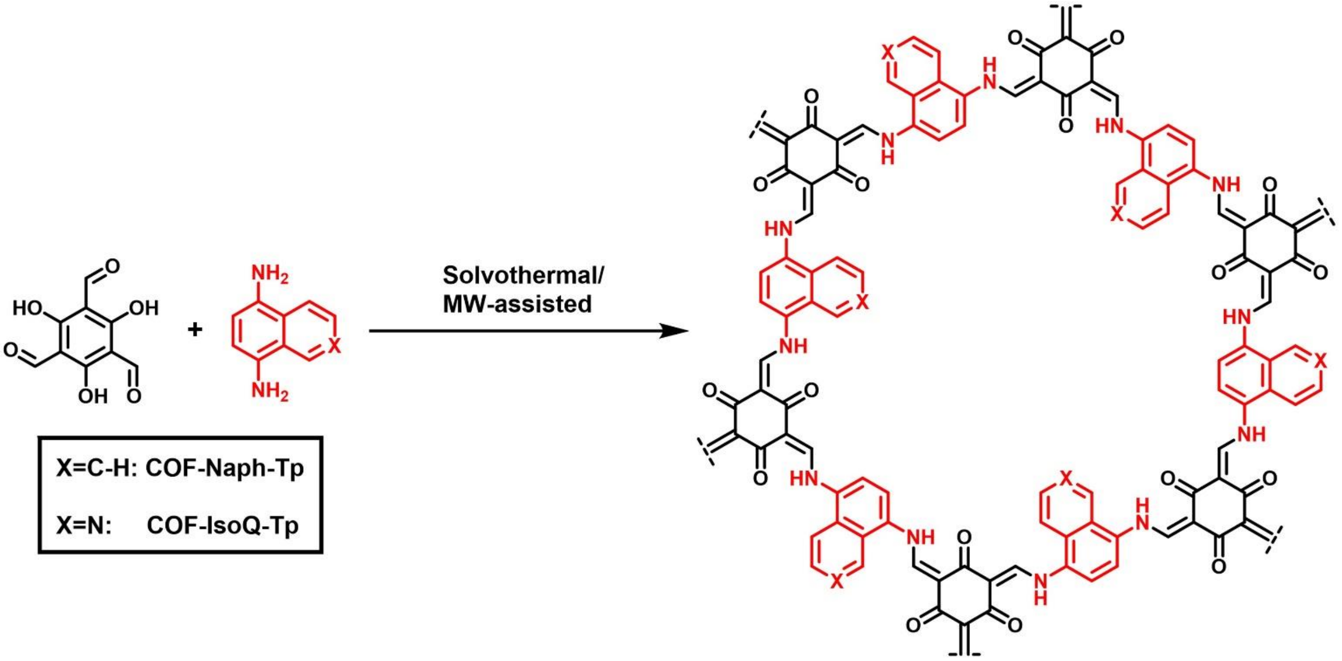

Scheme S3. Synthetic scheme of COF-IsoQ-Tp and COF-Naph-Tp.

General Solvothermal Synthesis

A Schlenk tube was charged with Tp (31.5 mg, 0.15 mmol), corresponding diamine [5,8-diaminoisoquinoline (35.8 mg, 0.225 mmol) for COF-IsoQ-Tp; 1,4-diaminonaphtalene (35.8 mg, 0.225 mmol) for COF-Naph-Tp], 2.25 mL of mesitylene, 2.25 mL of 1,4- dioxane, 0.3 mL of 6 M aqueous acetic acid under N_2 atmosphere. The mixture was purged with nitrogen for 10 minutes and then sonicated for 30 minutes to get a homogenous dispersion. The closed tube was heated at 120°C for 3 days. The precipitate was then recovered by filtration and washed with THF, DMF, and DCM. The powders collected were soaked in acetone and then dried at 120°C overnight.

General Microwave Assisted Synthesis

A MW tube was charged with Tp (31.5 mg, 0.15 mmol), corresponding diamine [5,8-diaminoisoquinoline (35.8 mg, 0.225 mmol) for COF-IsoQ-Tp; 1,4-diaminonaphtalene (35.8 mg, 0.225 mmol) for COF-Naph-Tp], 2.25 mL of mesitylene, 2.25 mL of 1,4- dioxane, under N_2 atmosphere. The mixture was purged with nitrogen for 10 minutes and then sonicated for 2 hours to get a homogeneous dispersion. 0.3 mL of 6 M acetic acid was added, and the dispersion was heated at 100°C (ca. 50 W microwave irradiation) for 1.5 hours. The precipitate was then recovered by filtration and washed with THF, DMF, and DCM. The powders collected were soaked in acetone and then dried at 120°C overnight.

S2.5: Electrodes Fabrication

FTO glass sheet was cut in a rectangular shape (~0.5x4 cm) and its surface was cleaned by 1 h ultrasonication in Alconox, followed by 1 h ultrasonication in deionized water, and finally 1 h ultrasonication in isopropanol. Two polished FTO electrodes were then immersed in the MW-assisted synthesis dispersion before sonication (see Above) using a 1:1 mixture of Mesitylene/Dioxane or 1,2-dichlorobenzene/butanol as solvent. MW synthesis is then carried out, as usual, to allow the direct growth of the COFs onto the electrodes. After completing the procedure, the COF-modified electrodes are thoroughly washed with THF. Finally, the material on the surface was partially removed to afford an active surface of 1 cm^2.

S3: Supporting Figures

S3.1: Benchmarking of state-of-the-art COF photocatalysts HER activity

Table S1. Photocatalytic performance comparison between COF-IsoQ-Tp, COF-Naph-Tp and similar material present in previous literature reports.

	% Pt	PtNP diameter (nm)	SED	Illumination	AQE	HER (μmol g^{-1} h^{-1})	Reference
**COF-IsoQ-Tp**	4	1.9-3.2	Ascorbic Acid	≥400 nm	0.2 % (456 nm)	11300	This Work
**COF-Naph-Tp**	4	2.0-3.1	Ascorbic Acid	≥400 nm	-	14	This Work
**Tp-COF**	6	~2.3	Ascorbic Acid	≥420 nm	-	265	[8]
**Pt-PVP-TPCOF**	6	~1.7	Ascorbic Acid	≥420 nm	0.4 % (475 nm)	8420	[8]
**TpPa COF NO_2**	3	-	Sodium Ascorbate	≥420 nm	-	220	[9]
**TpPa COF**	3	-	Sodium Ascorbate	≥420 nm	-	1560	[9]
**TpPa COF $(CH3)_2$**	3	-	Sodium Ascorbate	≥420 nm	-	8330	[9]
**TP-COF**	8	-	Ascorbic Acid	≥420 nm	-	1600	[10]
**S-COF**	8	-	Ascorbic Acid	≥420 nm	-	4440	[10]
**FS-COF**	8	3.0	Ascorbic Acid	≥420 nm	3.2 % (420 nm)	10100	[10]
**COF-TpPa-1/ TiO_2**	-	-	Ascorbic Acid	≥420 nm	-	1370	[11]
**CO-f-H**	3	~2	Ascorbic Acid	AM 1.5	-	5030	[12]
**COF-F**	**3**	~3	Ascorbic Acid	AM 1.5	0.29% (500 nm)	10580	[12]
**COF-Cl**	**3**	~2	Ascorbic Acid	AM 1.5	-	5840	[12]
**BDF-TAPT-COF**	**8**	2.5	Ascorbic Acid	AM 1.5	7.8% (420 nm)	1390	[13]
**H2Por-DETH-COF**	**8**	3.5	TEOA	≥400 nm	-	80	[14]
**CoPor-DETH-COF**	**8**	3.5	TEOA	≥400 nm	-	25	[14]
**NiPor-DETH-COF**	**8**	3.5	TEOA	≥400 nm	-	211	[14]
**ZnPor-DETH-COF**	**8**	3.5	TEOA	≥400 nm	0.32% (450 nm)	413	[14]
**COF-JLU-35**	**1**	1.5	Ascorbic Acid	≥420 nm	3.21 % (500 nm)	70800	[15]
**COF-JLU-36**	**1**	1.5	Ascorbic Acid	≥420 nm	-	24900	[15]
**BTT-BPy-COF**	**5**	1.8	Ascorbic Acid	≥420 nm	-	6800	[16]
**BTT-BPy-PCOF**	**5**	1.8	Ascorbic Acid	≥420 nm	3.72% (500 nm)	12300	[16]

S3.2: FT-IR Analysis

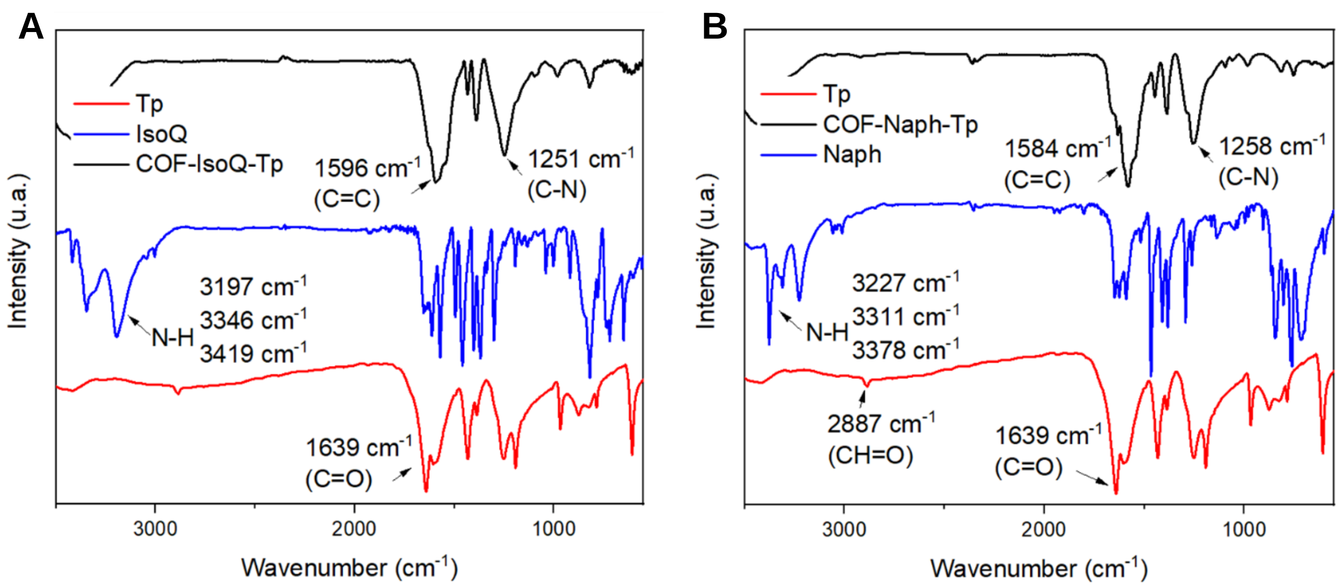

Figure S1. (A) FT-IR of 2,4,6-Triformylphloroglucinol (black trace), 5,8-diaminoisoquinoline (blue trace) and COF-IsoQ-Tp (red trace); (B) FT-IR of 2,4,6-Triformylphloroglucinol (black trace), 1,4-diaminonaphtalene (blue trace) and COF-Naph-Tp (red trace).

S3.3: Elemental Analysis

Table S2. Elemental analysis of COF-IsoQ-Tp and COF-Naph-Tp. Theoretical values are calculated for 100 % conversion of the monomers.

**Material**	**Theoretical**				**Experimental**			
	C / wt-%	H / wt-%	N / wt-%	C/N molar ratio	C / wt-%	H / wt-%	N / wt-%	C/N molar ratio
**COF-IsoQ-Tp**	68.4	3.4	16.0	4.3	63.66	3.99	15.26	4.2
**COF-Naph-Tp**	73.0	3.8	10.6	6.9	69.40	4.22	10.08	6.9

S3.4: PXRD Analysis

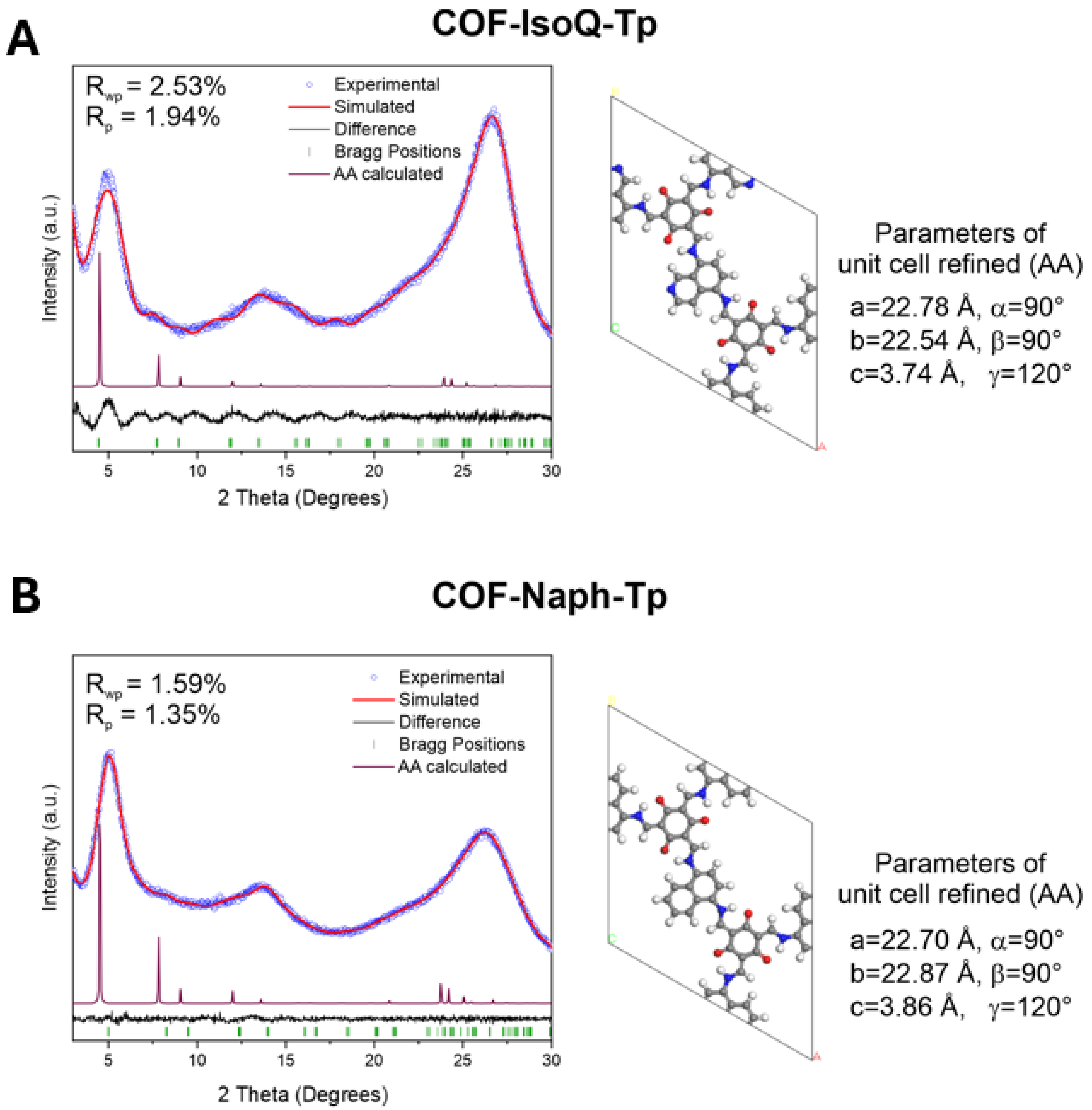

Figure S2. A) PXRD pattern of COF-IsoQ-Tp (open, blue circles), Pawley refined profile (red trace), and calculated XRD pattern for the idealized AA stacking (deep red trace). The unit cell and its parameters for the idealized AA stacking model are reported on the right side. B) PXRD pattern of COF-Naph-Tp (open, blue circles), Pawley refined profile (red trace), and calculated XRD pattern for the idealized AA stacking (deep red trace). The unit cell and its parameters for the idealized AA stacking model are reported on the right side

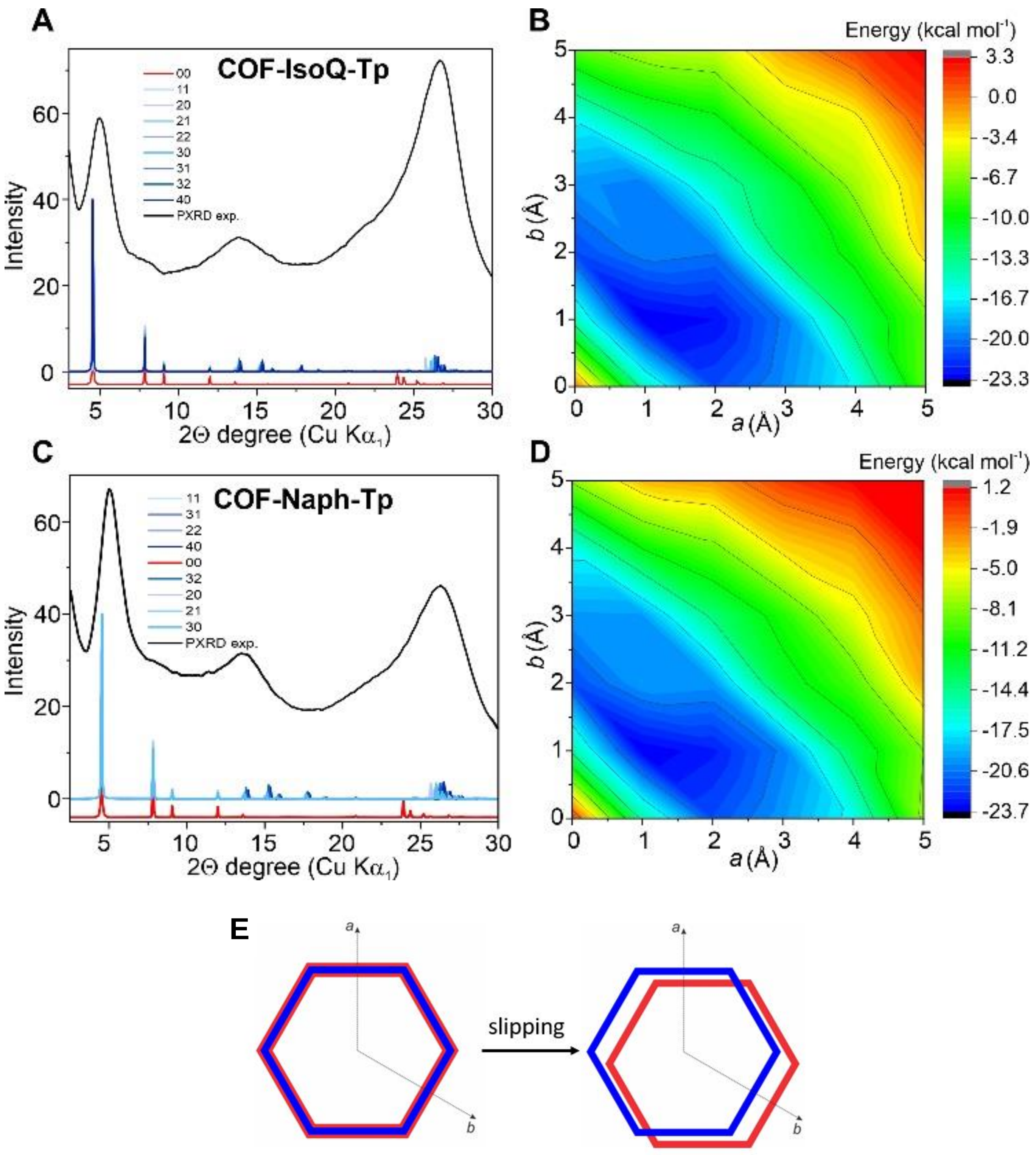

Figure S3. PXRD characterization of A) COF-IsoQ-Tp and C) COF-Naph-Tp: experimental patterns (black line) against simulated patterns of AA configuration for the most stable computed structures (blu lines) and the eclipsed configuration (red line). Contour plot showing the energy variation of the COF structure with eclipsed AA arrangement as a function of slipping along a and b directions for B) COF-IsoQ-Tp and D) COF-Naph-Tp. E) Model structure depicting the axis (a, b) considered for the slipping directions.

S3.5: BET Analysis

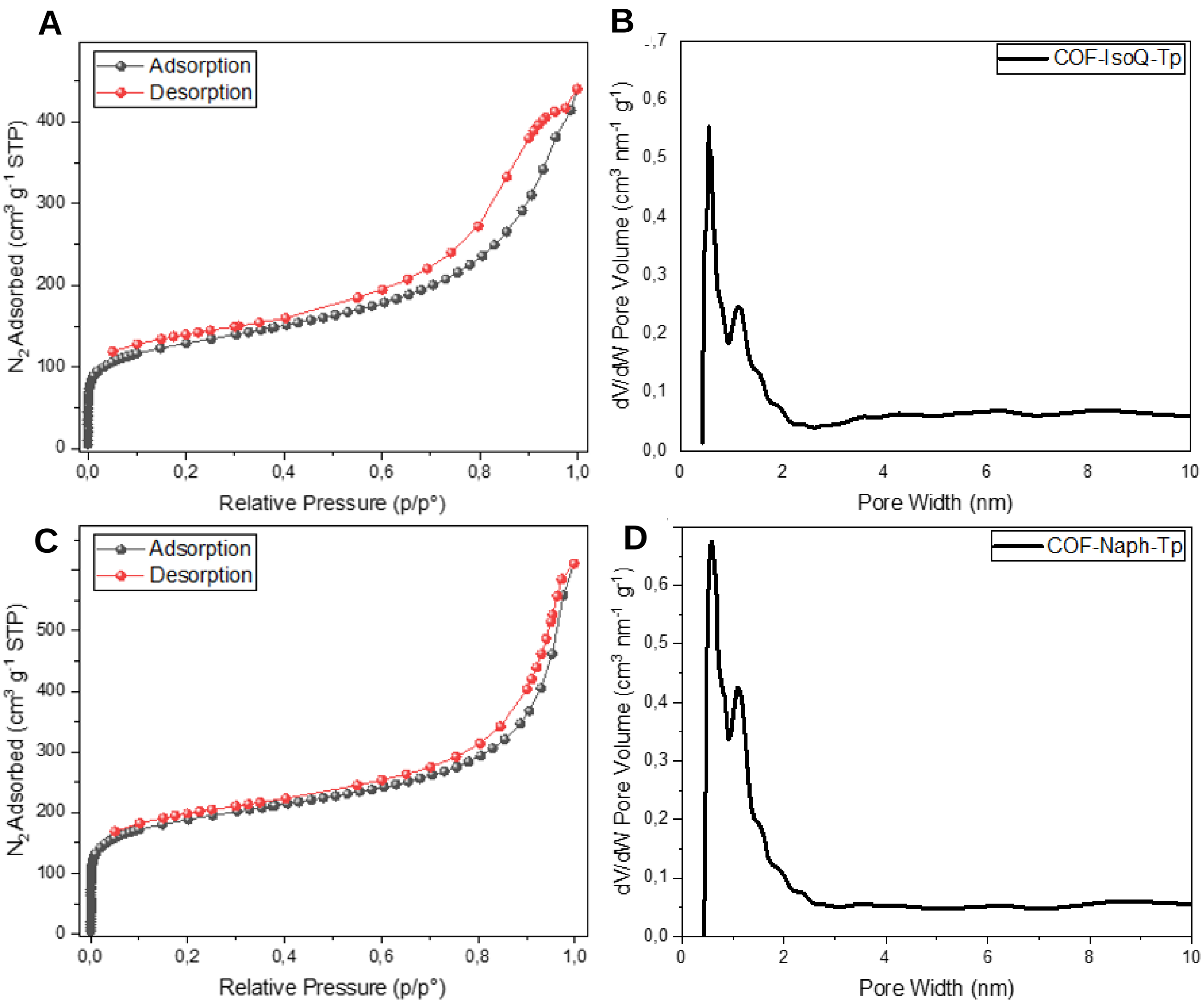

Figure S4. (A) N_2 adsorption/desorption isotherms for COF-IsoQ-Tp; (B) Pore size distribution for COF-IsoQ-Tp. (C) N_2 adsorption/desorption isotherms for COF-Naph-Tp; (D) Pore size distribution for COF-Naph-Tp.

S3.6: X-Ray Photoelectron Spectroscopy (XPS)

COF-IsoQ-Tp and COF-Naph-Tp

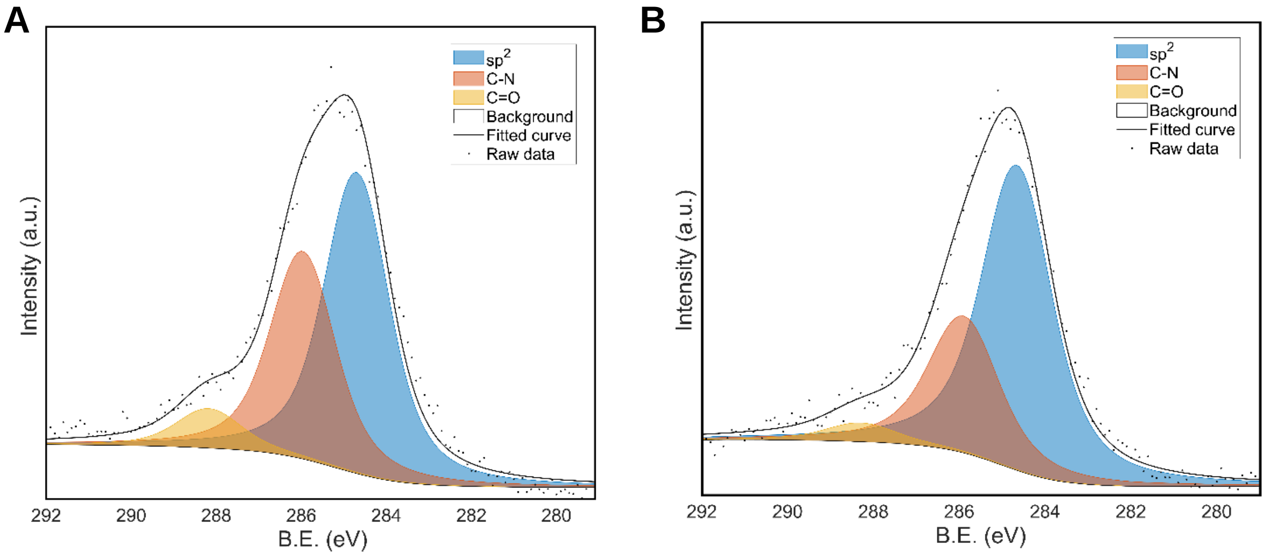

Figure S5. C 1s XPS region of (A) COF-IsoQ-Tp and (B) COF-Naph-Tp.

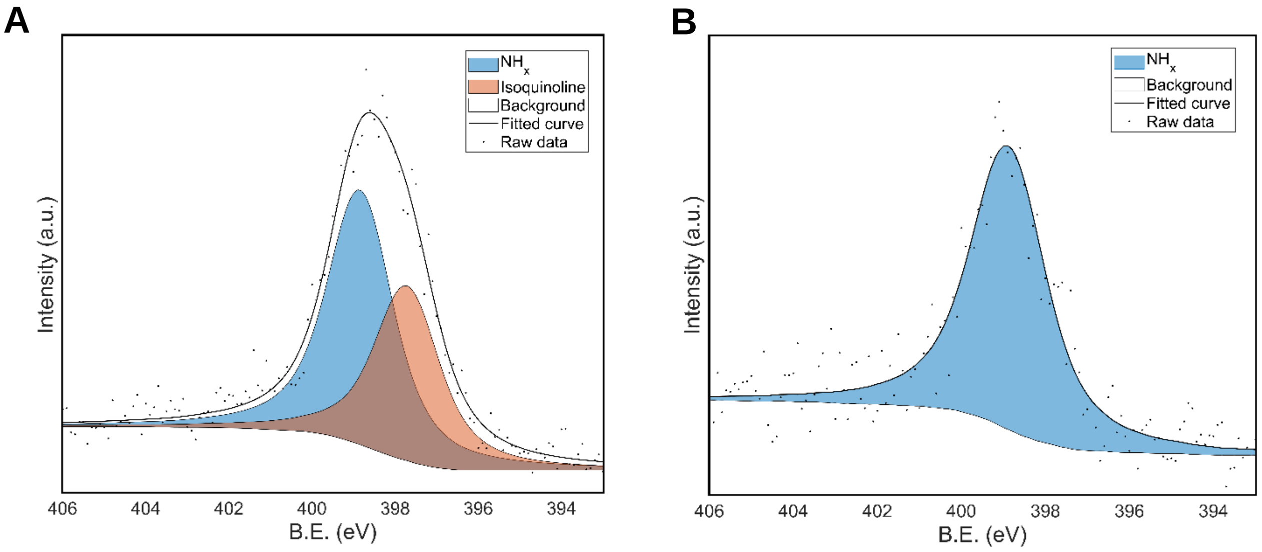

Figure S6. N 1s XPS region of (A) COF-IsoQ-Tp and (B) COF-Naph-Tp.

Pt@COF-IsoQ-Tp and Pt@COF-Naph-Tp

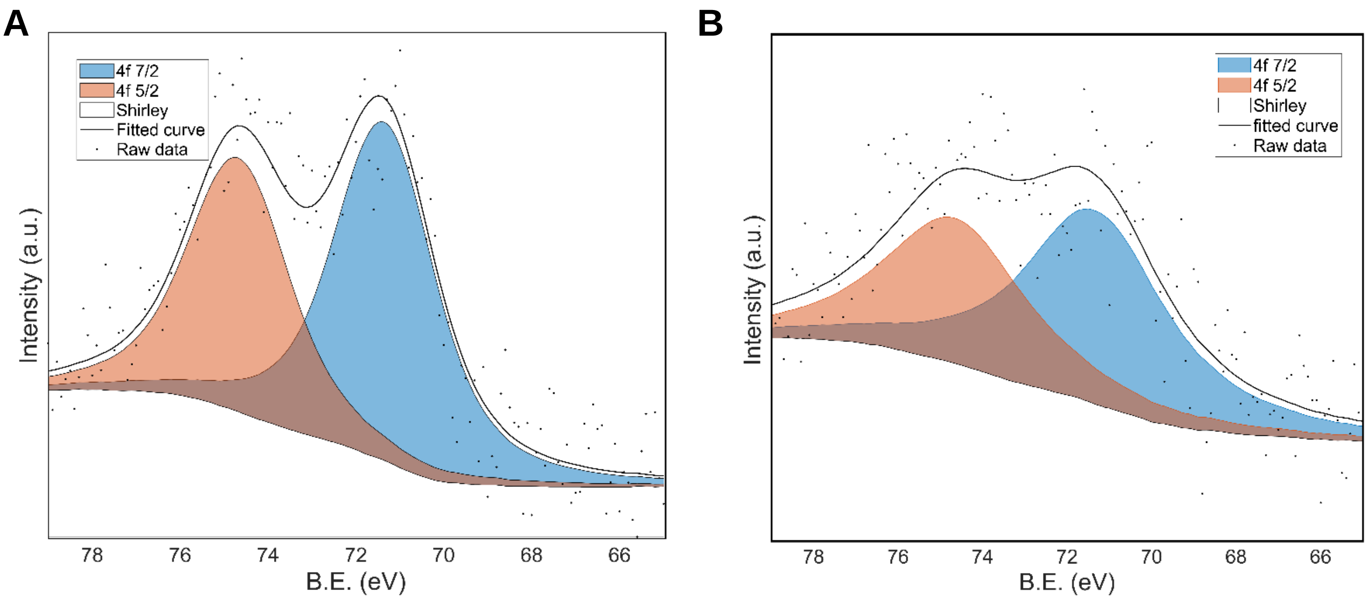

Figure S7. Pt 4f XPS region of (A) Pt@COF-IsoQ-Tp and (B) Pt@COF-Naph-Tp.

S3.7: Thermogravimetric Analysis (TGA)

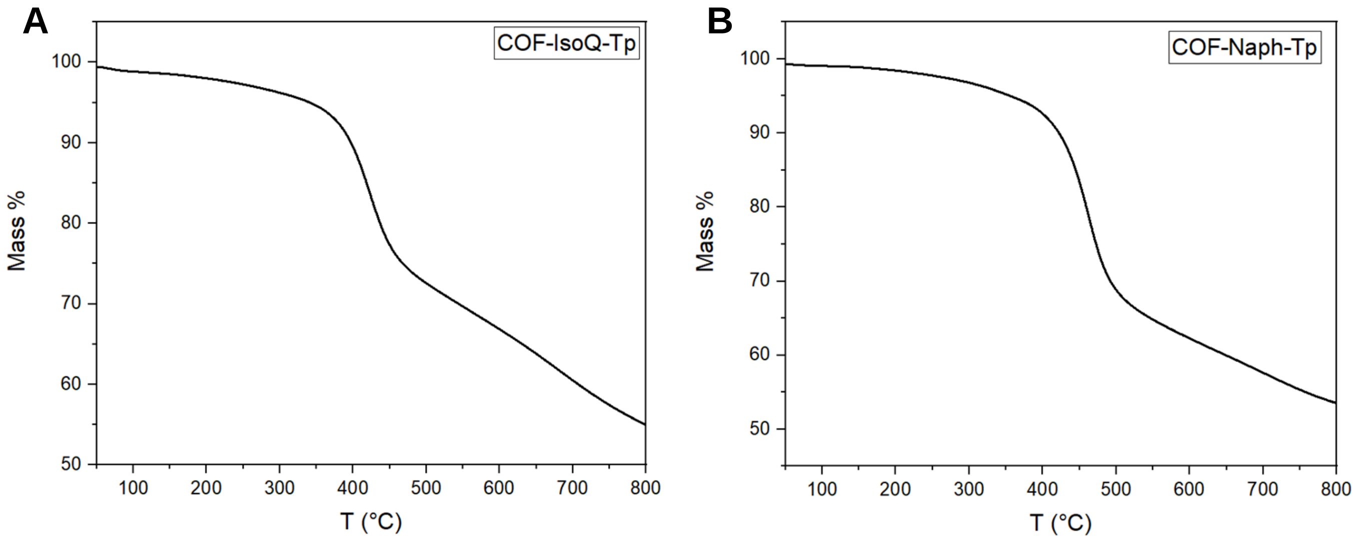

Figure S8. Thermogravimetric analysis of (A) COF-IsoQ-Tp and (B) COF-Naph-Tp.

S3.8: Diffuse Reflectance

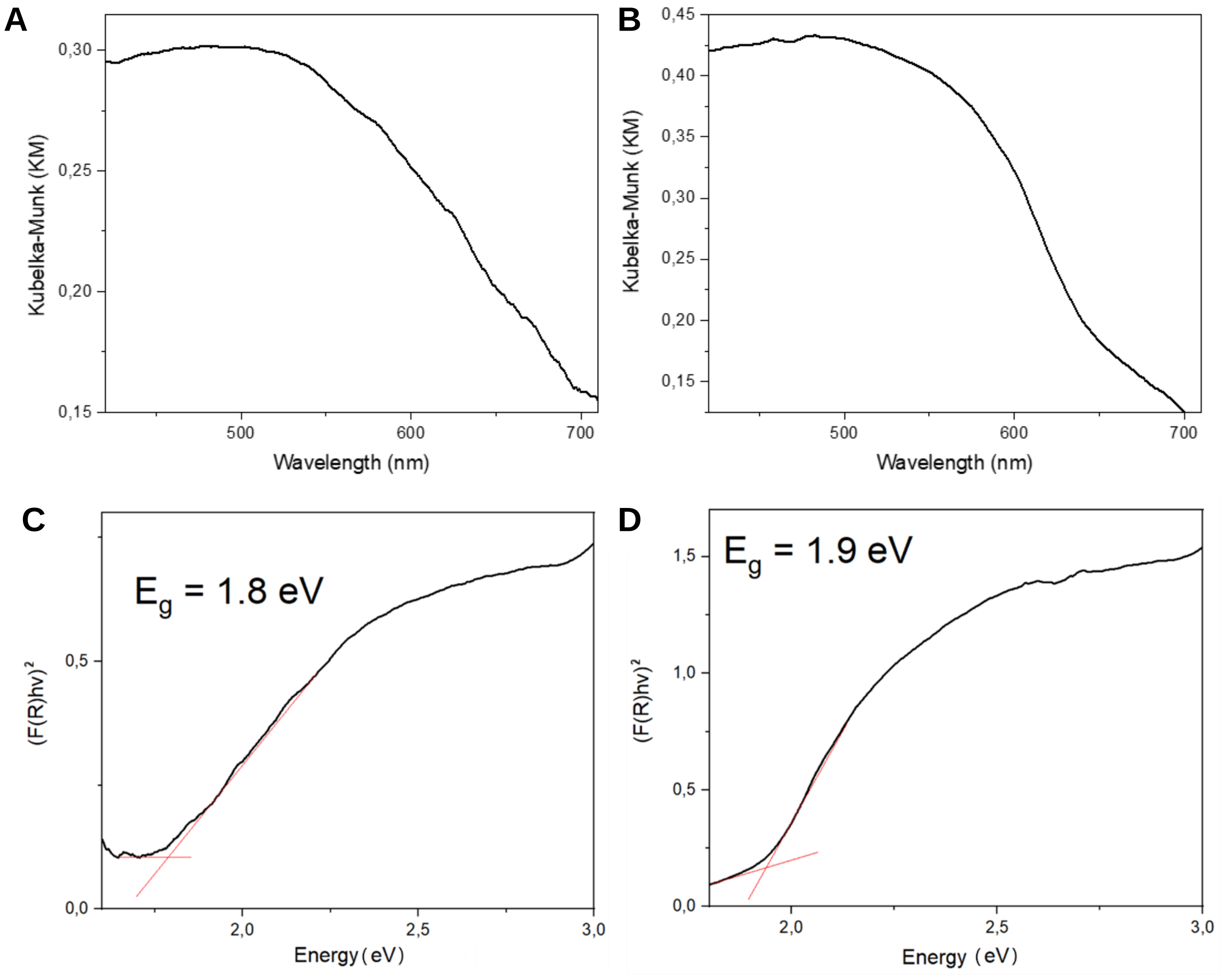

Figure S9. Diffuse reflectance spectra of (A) COF-IsoQ-Tp and (B) COF-Naph-Tp. Tauc plots of (C) COF-IsoQ-Tp and (D) COF-Naph-Tp.

S3.9: Photoluminescence

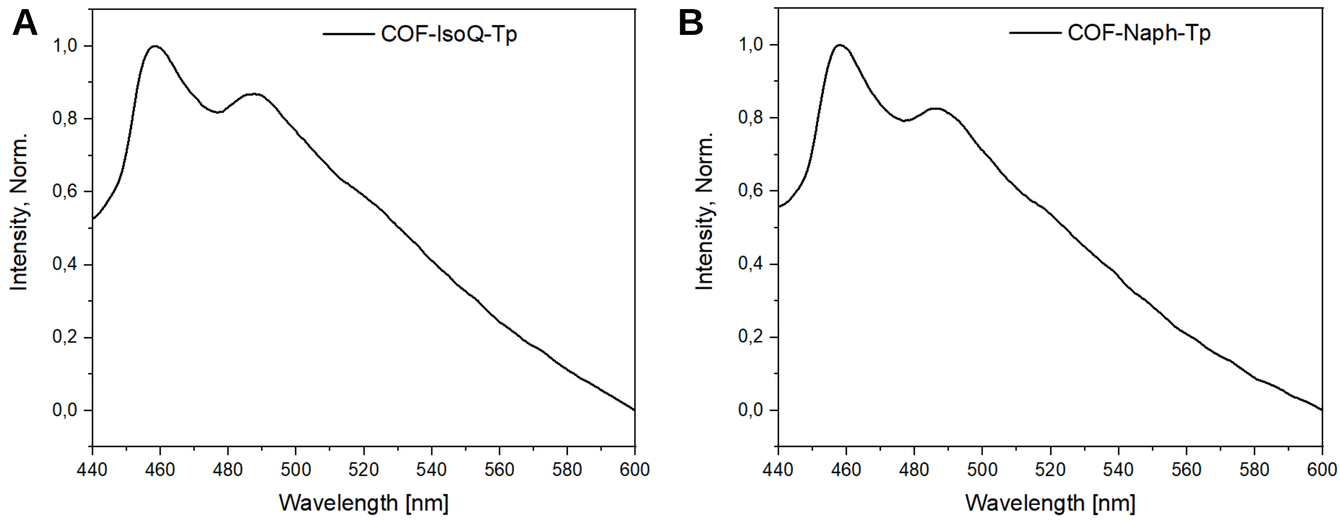

Figure S10. Photoluminescence spectra (λ_{ex}=420 nm) of (A) COF-IsoQ-Tp and (B) COF-Naph-Tp.

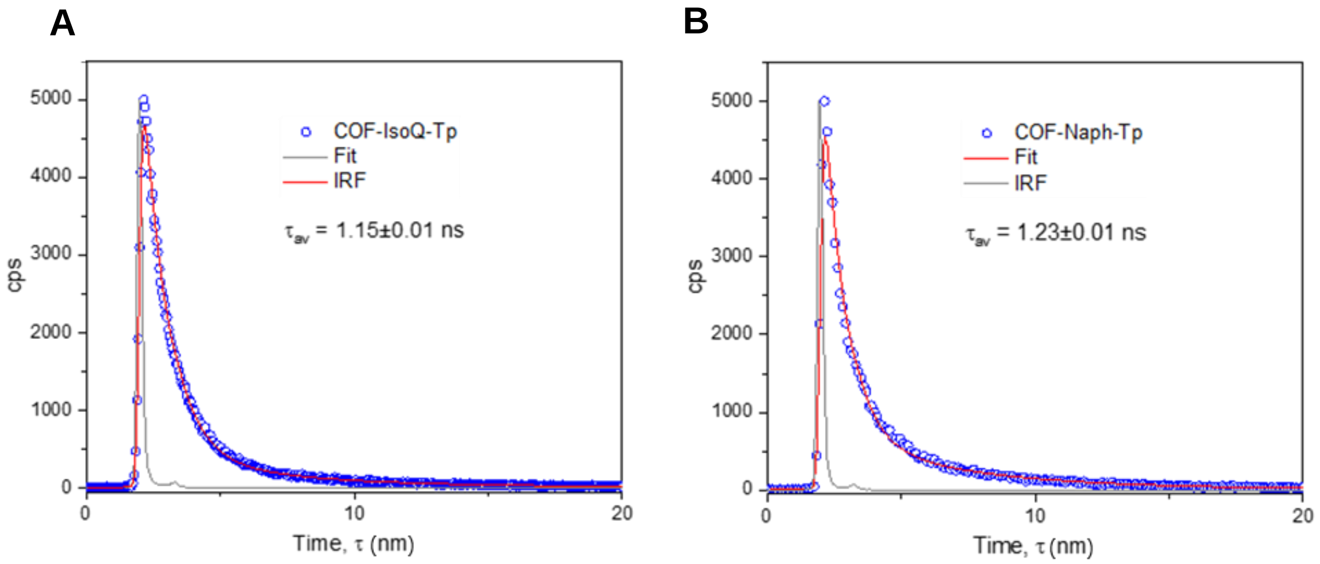

Figure S11. Time-dependent fluorescence of (A) COF-IsoQ-Tp, (B) COF-Naph-Tp.

Table S3. Measured lifetimes and weighted average lifetimes for the two COFs.

	τ_1 [ns]	τ_2 [ns]	$\langle\tau\rangle$ [ns]
COF-IsoQ-Tp	0.86, (92%)	4.59, (8%)	1.15 ± 0.01
COF-Naph-Tp	0.76, (88%)	4.76, (12%)	1.23 ± 0.01

S3.10: Mott-Schottky Spectroscopy

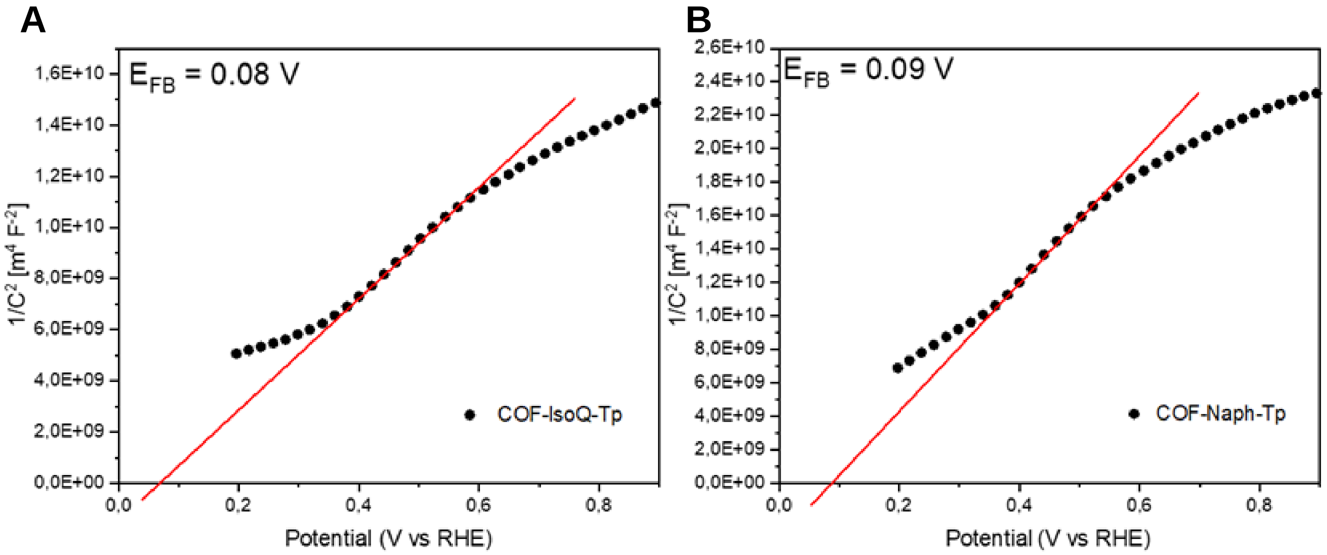

Figure S12. Mott-Schottky plots of (A) COF-IsoQ-Tp and (B) COF-Naph-Tp electrodes at 1000 Hz.

S3.11: Electrode Characterization

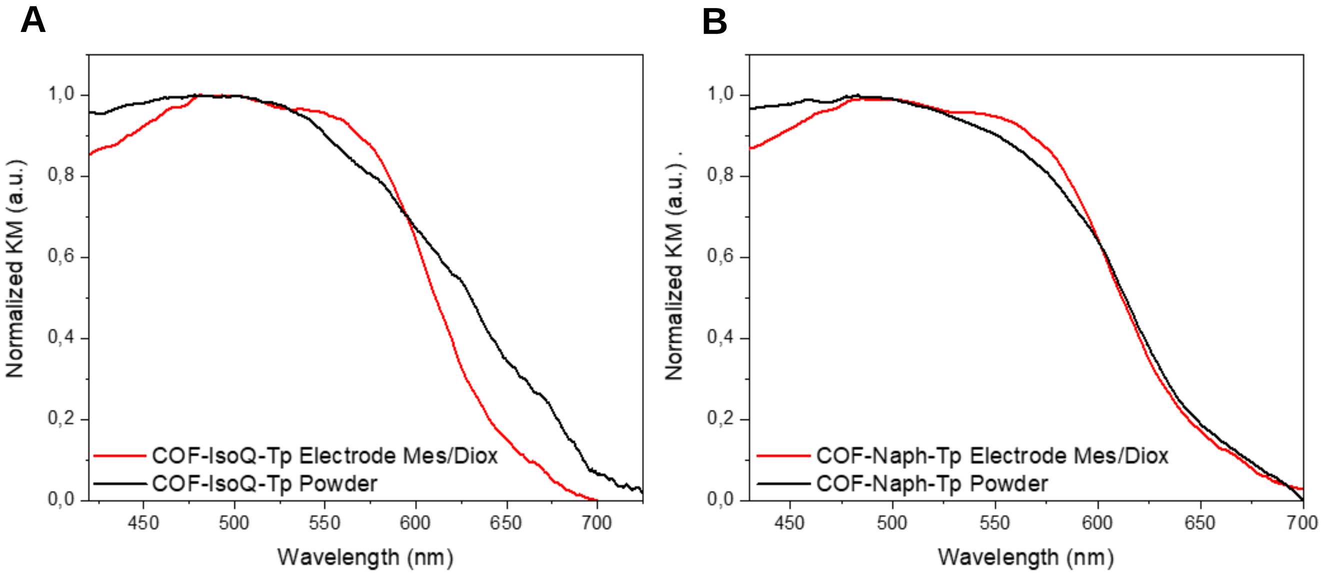

Figure S13. Diffuse reflectance spectra of COF powder (black trace) and COF@FTO synthesized from 1:1 mixture of Mesitylene and Dioxane (red trace) for (A) COF-IsoQ-Tp and (B) COF-Naph-Tp.

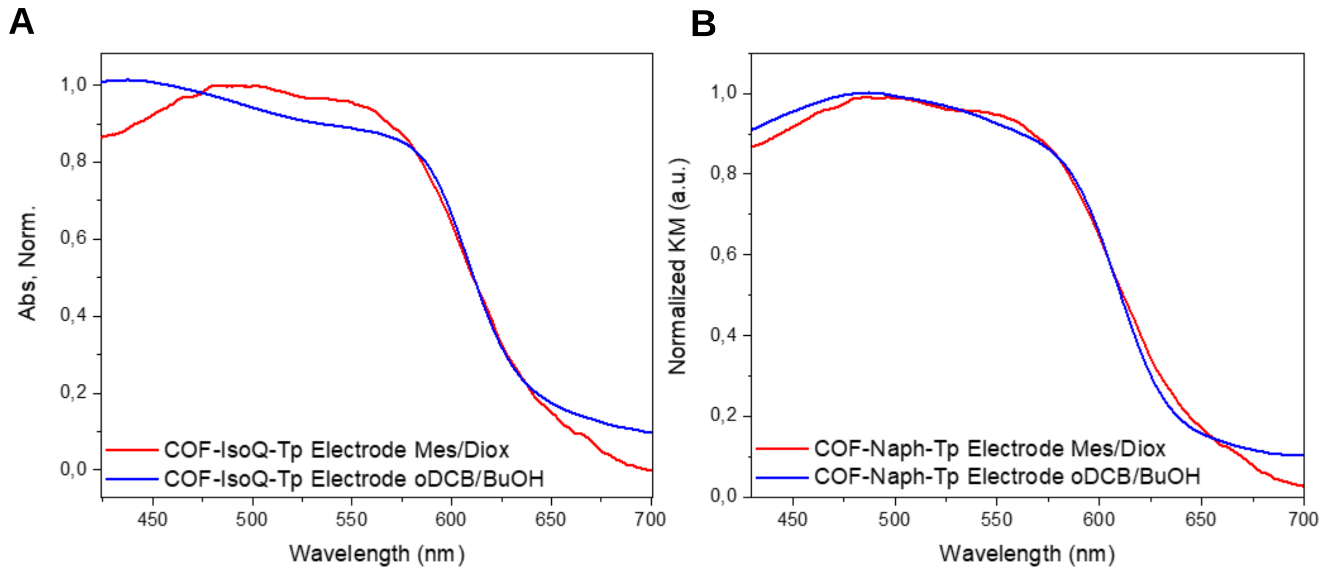

Figure S14. Diffuse reflectance spectra of COF@FTO synthesized from 1:1 mixture of Mesitylene and Dioxane (red trace) and COF@FTO synthesized from 1:1 mixture of 1,2-dichlorobenzene and butanol (blue trace) for (A) COF-IsoQ-Tp and (B) COF-Naph-Tp.

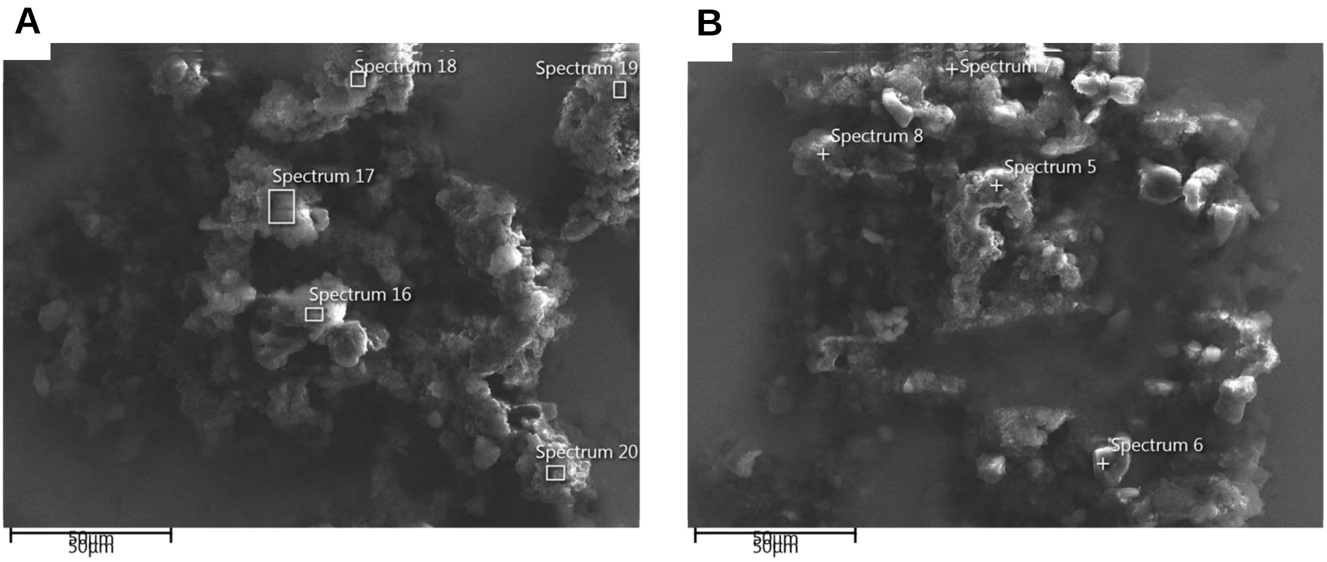

Figure S15. SEM-EDX experiments on (A) COF-IsoQ-Tp@FTO and (B) COF-Naph-Tp@FTO.

Table S4 SEM-EDX results for: Left) COF-IsoQ-Tp and Right) COF-Naph-Tp, compared with theoretical elemental analysis results.

**COF-IsoQ-Tp**	Weight %	Standard Deviation	Expected Values
C	64	2	68.4
N	18	1	16.0
O	18	1	12.2

**COF-Naph-Tp**	Weight %	Standard Deviation	Expected Values
C	77	4	73.0
N	8	2	10.6
O	14	2	12.6

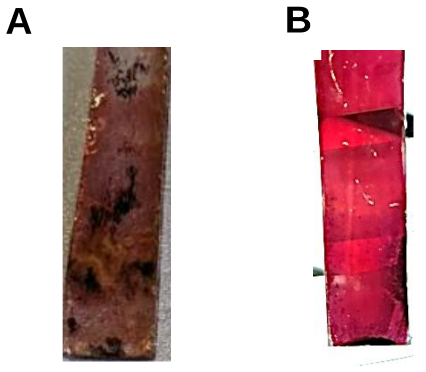

Figure S16. (A) COF-IsoQ-Tp@FTO electrode synthesized by microwave-assisted synthesis employing Mesitylene/Dioxane 1:1 mixture as solvents. (B) COF-IsoQ-Tp@FTO electrode synthesized by microwave-assisted synthesis employing Butanol/1,2-dichlorobenzene 1:1 mixture as solvents.

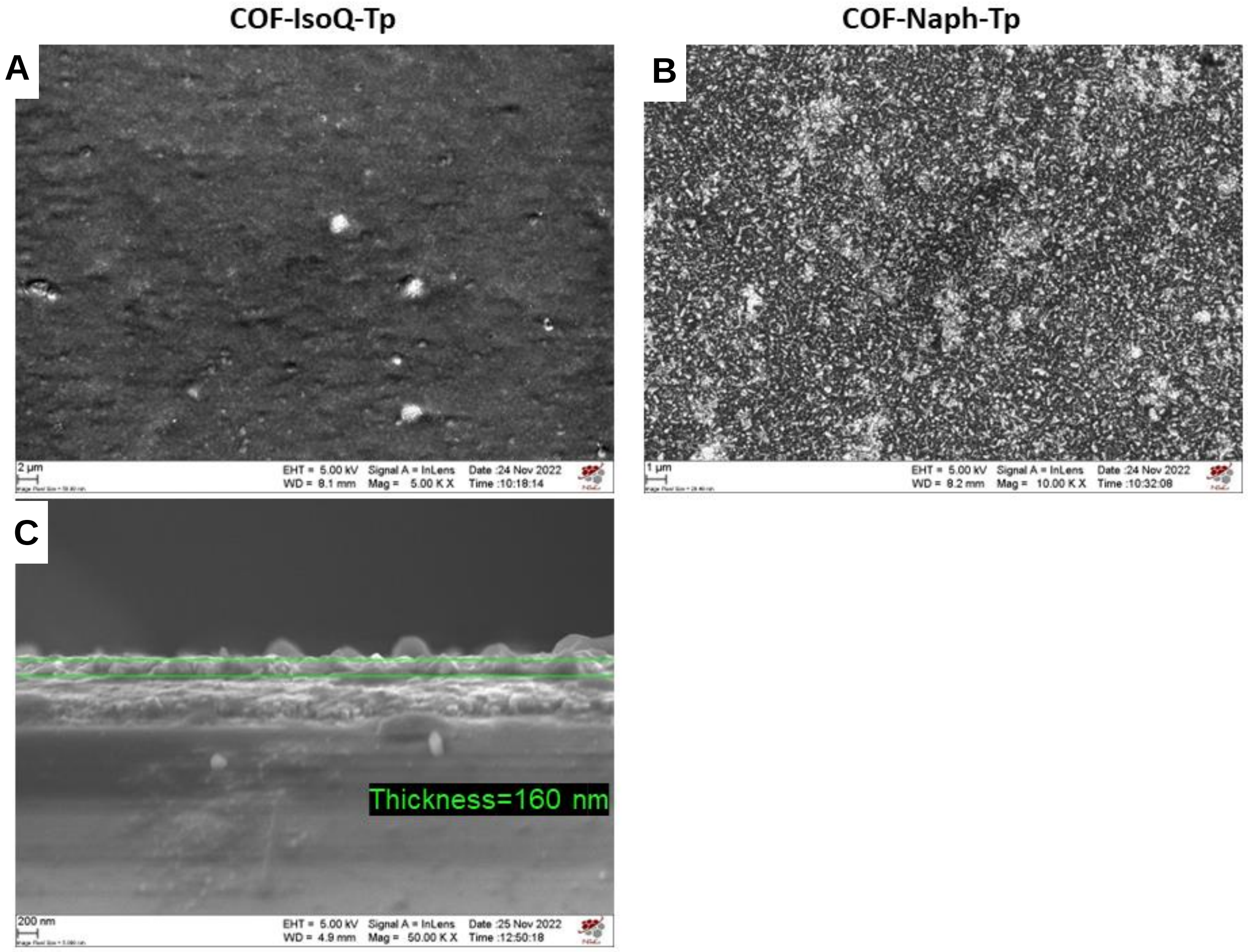

Figure S17. Top view SEM images on (A) COF-IsoQ-Tp@FTO and (B) COF-Naph-Tp@FTO. Cross-sectional view of (C) COF-IsoQ-Tp@FTO and measured COF layer thickness. COF-Naph-Tp layer thickness could not be measured because of the inhomogeneity of the film.

S3.12: Photoelectrochemical Experiments

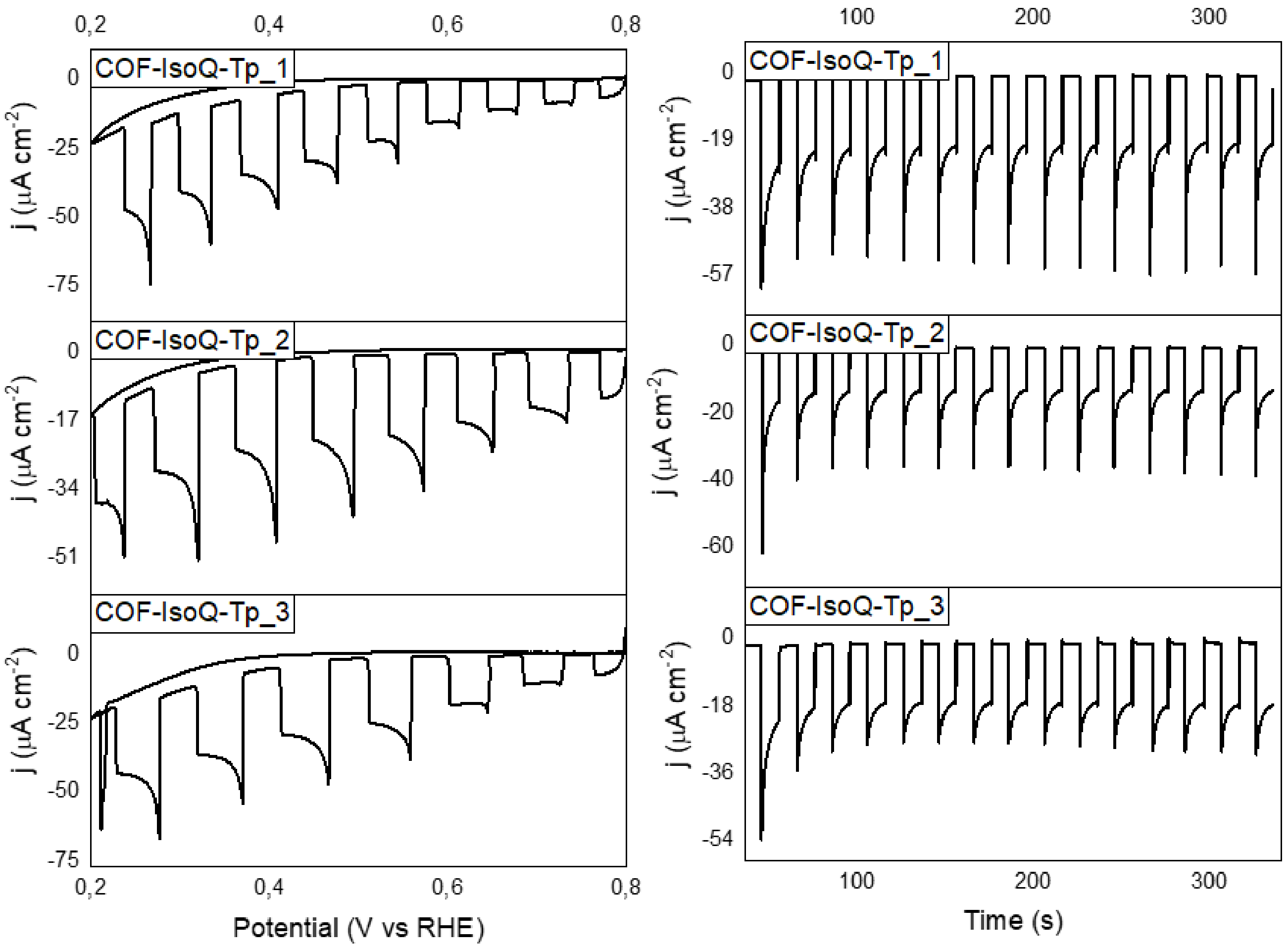

Figure S18. Left: Chopped light for COF-IsoQ-Tp@FTO on three different batches. Right: Chopped light chronoamperometries plot for COF-IsoQ-Tp@FTO (applied bias 0.4 V vs RHE). The COF film was obtained by directly growing the material in situ from a mixture of 1,2-dichlorobenzene and butanol. The photoelectrochemical experiments were carried out in sodium sulfate Na_2SO_4 0.1 M, pH 7, the measurements were recorded at 200 mW cm^{-2}.

Table S5. Photocurrent Densities obtained at three different Applied potentials. The photoelectrochemical experiments were carried out in sodium sulfate Na_2SO_4 0.1 M, pH 7, the measurements were recorded at 200 mW cm^{-2}

Applied Potential (V vs RHE)	J (μA cm^{-2})	Standard Deviation
0.25	-29	3
0.40	-16	4
0.68	-12	3

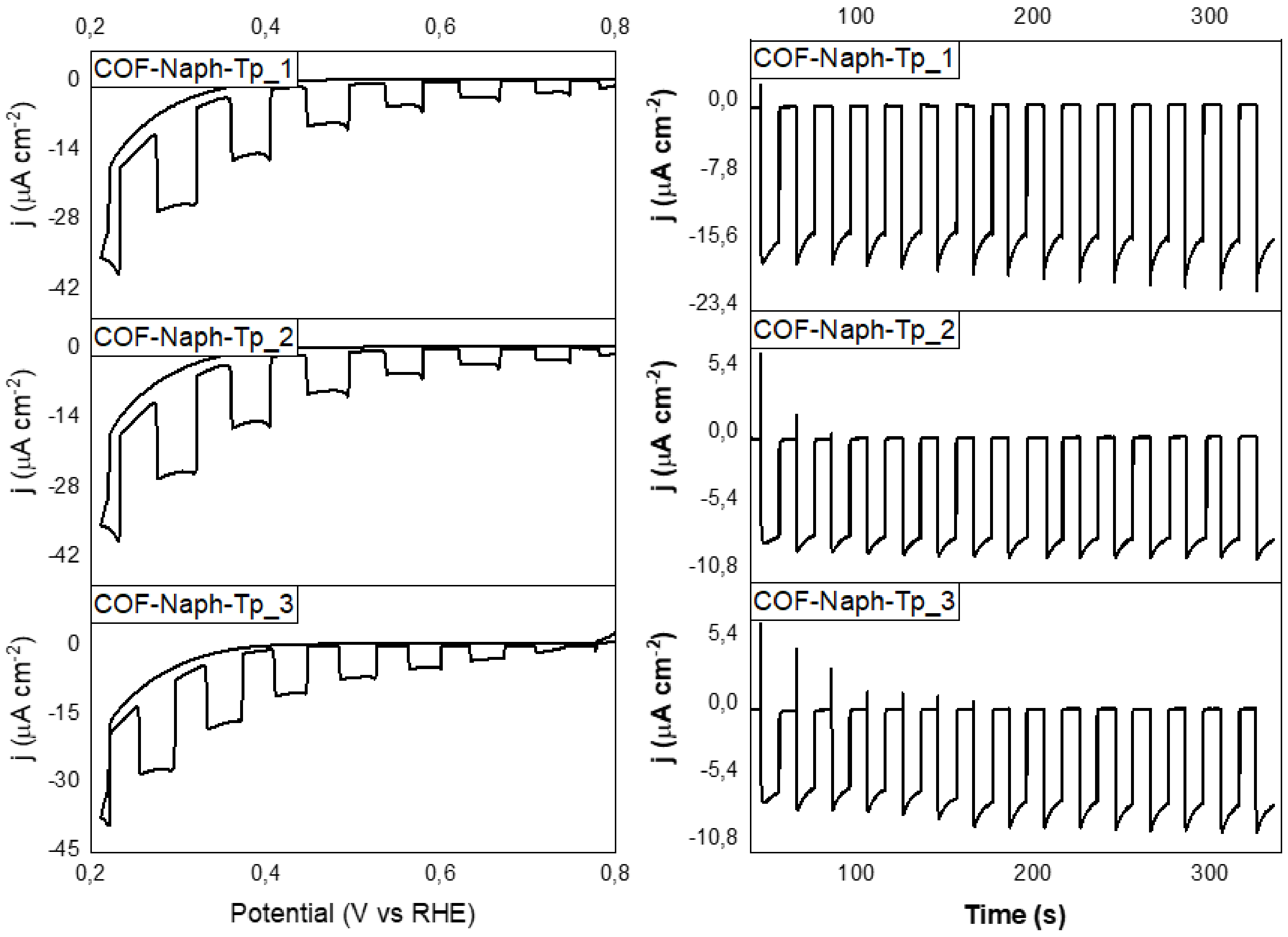

Figure S19. Left: Chopped light for COF-Naph-Tp@FTO on three different batches. Right: Chopped light chronoamperometries plot for COF-Naph-Tp@FTO (applied bias 0.4 V *vs* RHE). The COF film was obtained by directly growing the material in situ from a mixture of 1,2-dichlorobenzene and butanol. The photoelectrochemical experiments were carried out in sodium sulfate Na_2SO_4 0.1 M, pH 7; the measurements were recorded at 200 mW cm^{-2}.

Table S6. Photocurrent Densities obtained at three different Applied potentials. The photoelectrochemical experiments were carried out in sodium sulfate Na_2SO_4 0.1 M, pH 7, the measurements were recorded at 200 mW cm^{-2}.

Applied Potential (V *vs* RHE)	J (µA cm^{-2})	Standard Deviation
0.25	–22	5
0.40	–12	5
0.68	–4	2

Table S7 Summary of reported Photoelectrochemical performance of COF-based electrodes.

	**Potential (V vs RHE)**	**Illumination**	**j (μA cm^{-2})**	**Photocurrent Stability**	**Reference**
COF-IsoQ-Tp	0.4	AM 1.5G<br>200 mW cm^{-2}	-16	300s<br>20% loss	This Work
COF-Naph-Tp	0.4	AM 1.5G<br>200 mW cm^{-2}	-12	300s<br><5%	This Work
2D CCP-Th	0.3	AM 1.5G	-5.5	350 s<br>26% loss	[17]
2D CCP-BD	0.3	AM 1.5G	-2.8	350 s<br>10% loss	[17]
g-C18N3-COF	0.4	100 mW cm^{-2}<br>λ>420 nm	-25	350 s<br>30% loss	[18]
g-C33N3-COF	0.4	100 mW cm^{-2}<br>λ>420 nm	-8	350 s<br>15% loss	[18]
g-C54N6-COF	1.2	300 mW cm^{-2}<br>λ>420 nm	7	120 s<br><5% loss	[19]
g-C52N6-COF	1.2	300 mW cm^{-2}<br>λ>420 nm	5	120 s<br><5% loss	[19]
BDT-ETTA COF	0.4	100 mW cm^{-2}	-0.9	18000 s<br>8% loss	[20]
re-BT-COF	-	100 mW cm^{-2}<br>λ>420 nm	~-6	200 s<br><5% loss	[21]
A-TENPY-COF	-	AM 1.5G<br>100 mW cm^{-2}	-1.5	120 s<br>32% loss	[22]
A-TEBPY-COF	-	AM 1.5G<br>100 mW cm^{-2}	-6	120 s<br>45% loss	[22]
Bpy-sp2c-COF	0.5	300 W Xe<br>λ> 420 nm	~-0.5	200 s<br><5% loss	[23]
v-2D-COF-NO1	0.3	100 mW cm^{-2}<br>λ>420 nm	-18	1000 s<br><5% loss	[24]
v-2D-COF-NO2	0.3	100 mW cm^{-2}<br>λ>420 nm	-9.1	1000 s<br><5% loss	[24]

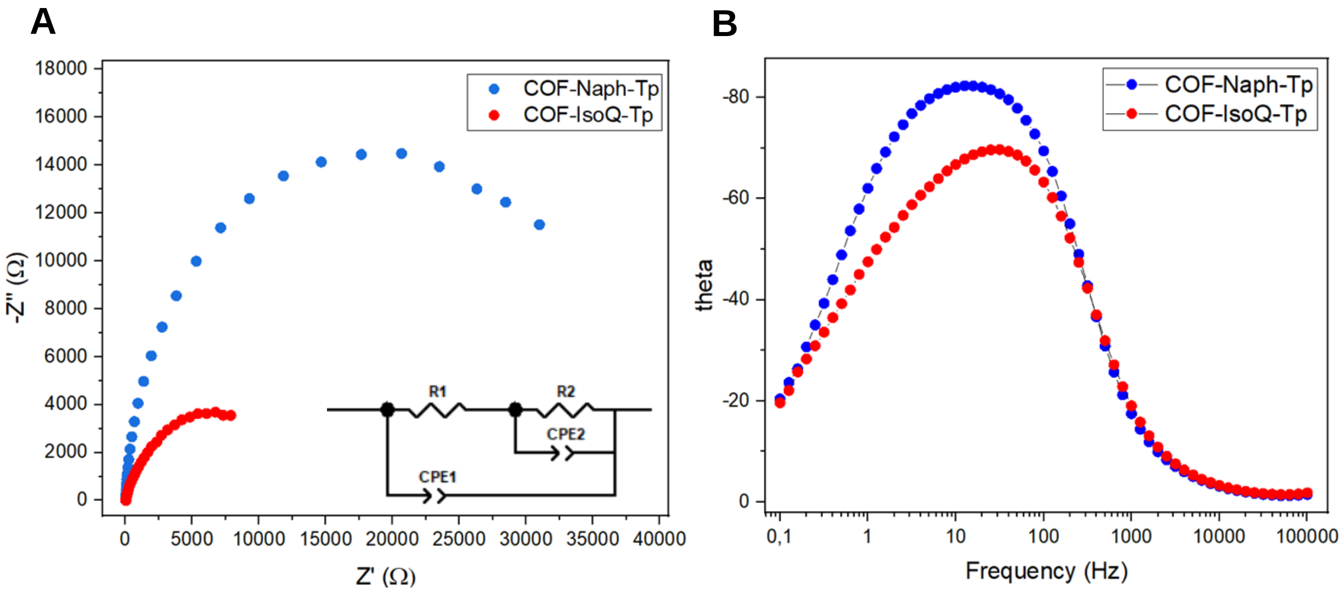

Figure S20. (A) Nyquist plot and (B) Bode phase plot for COF-IsoQ-Tp@FTO (red traces) and COF-Naph-Tp@FTO (blue traces). Registered at 0.4 V vs RHE in 0.1 M Na_2SO_4, pH 7 with solar simulator AM 1.5 G, 200 mW cm^{-2}.

In Figure S21 are reported the Nyquist plots acquired for the two materials along with the corresponding fitting. The analysis of the data using the classic Randles circuit (Figure S19 B) fails to yield an accurate fit.

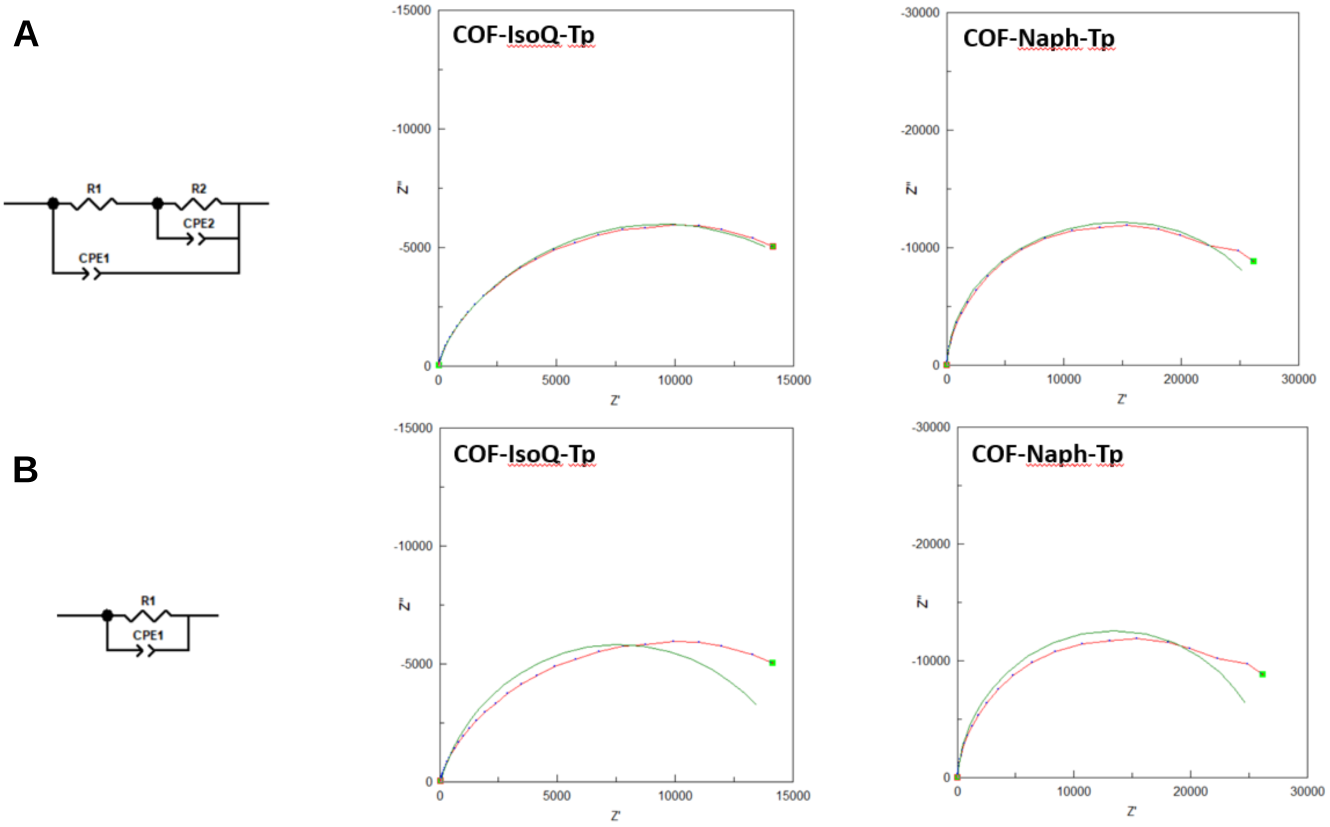

Figure S21. COF-IsoQ-Tp and COF-IsoQ-Tp experimental Nyquist plots (red trace) and curve fitting (green light) employing (A) a modified Randles circuit and (B) a classical Randles circuit.

Table S8. Summary of reported Photoelectrochemical impedance spectroscopy (PEIS) analysis performed on COF-based materials. Bottom: Randles circuit (a) and modified Randles circuits (b,c) employed as models for the reported systems

Material	Circuit	Resistance	Reference
COF-IsoQ-Tp	b	R1= 3±1 kΩ<br>R2= 16±4 kΩ	This Work
COF-Naph-Tp	b	R1= 21±2 kΩ<br>R2= 10±4 kΩ	This Work
BD-CON	a	65.910 kΩ	[25]
CYANO-CON	a	8.349 kΩ	[25]
TpDPP-COF	b	R1= 10.419 kΩ<br>R2= 1.338 kΩ	[26]
TpEtBr-COF	b	R1= 0.09731 kΩ<br>R2= 0.06744 kΩ	[26]
TpTab-COF	b	R1= 0.1016 kΩ<br>R2= 130.56 kΩ	[26]
TpTta-COF	b	R1= 0.1015 kΩ<br>R2= 145 kΩ	[26]
PTB7-Th:PDI-V BHJ	b	R1= 0.08-1 kΩ cm^{-2}<br>R2=0.01-0.1 kΩ cm^{-2}	[27]
Bipy-COF	c	-	[28]

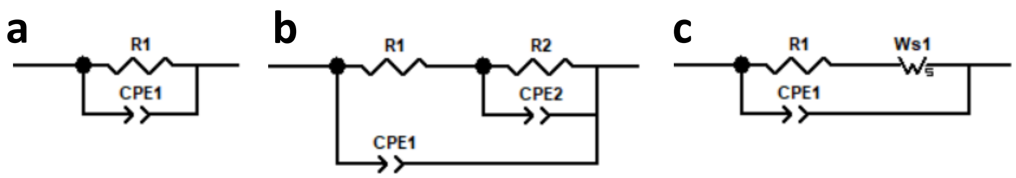

S3.13: Photocatalytic Hydrogen Evolution

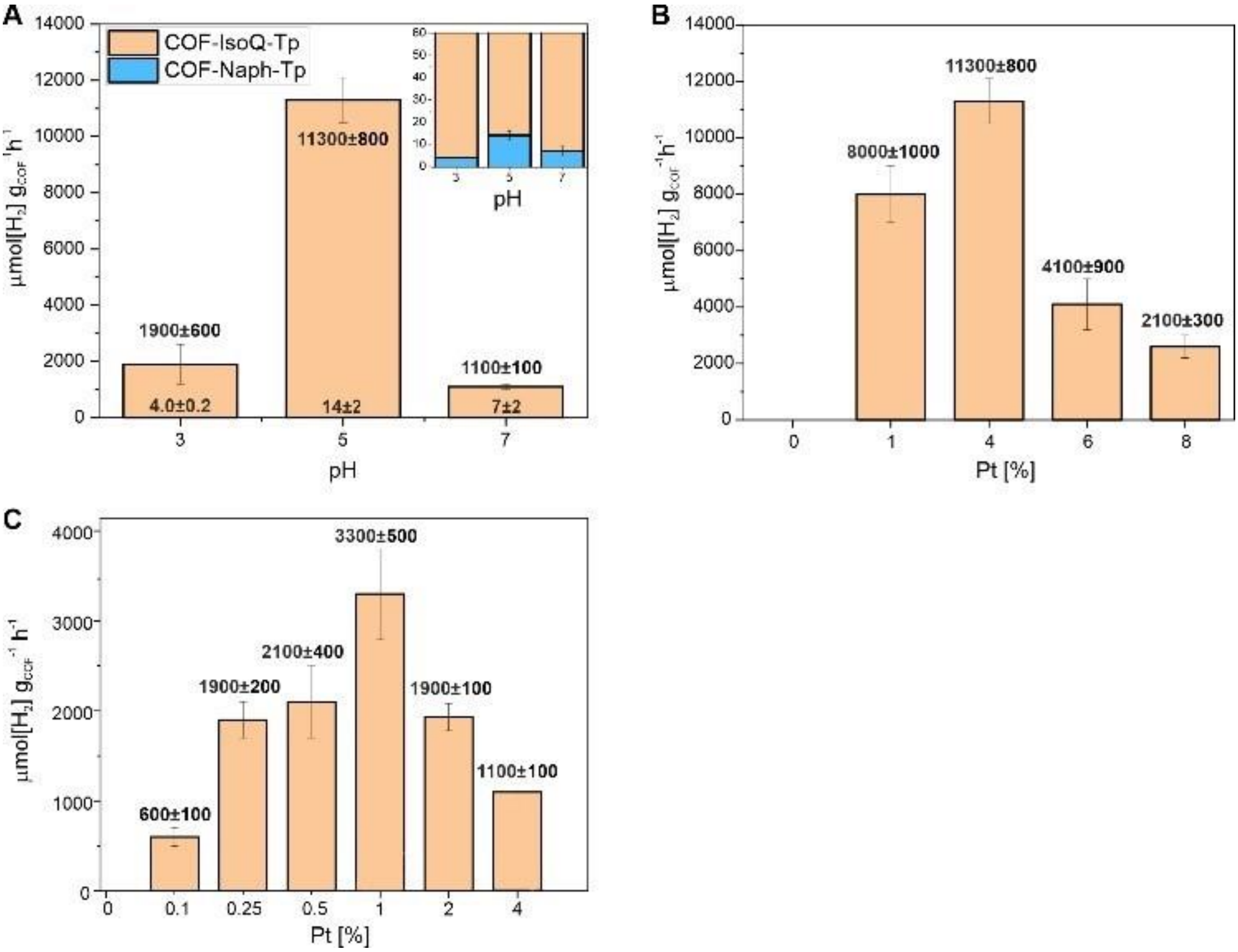

Figure S22. (A) Photocatalytic hydrogen evolution rate as a function of pH in presence of 4% platinum. (B) Photocatalytic hydrogen evolution rate as a function of platinum amount (%) at pH 5. (C) Photocatalytic hydrogen evolution rate as a function of platinum amount (%) at pH 7.

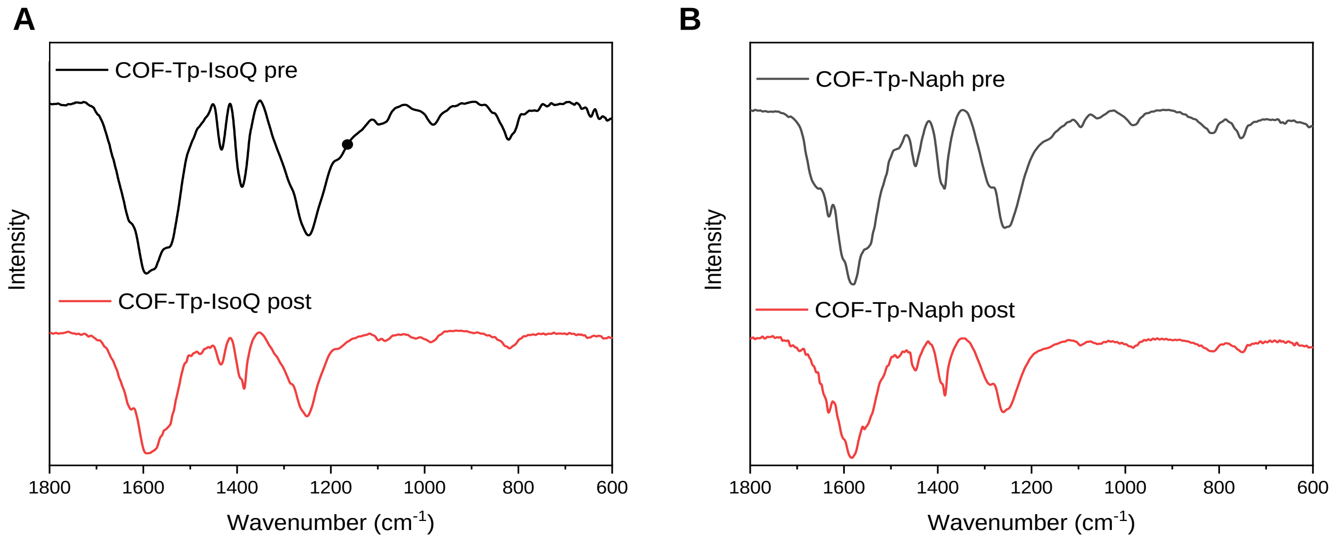

Figure S23. FT-IR spectra before (black trace) and after (red trace) photocatalysis for (A) COF-IsoQ-Tp and (B) COF-Naph-Tp.

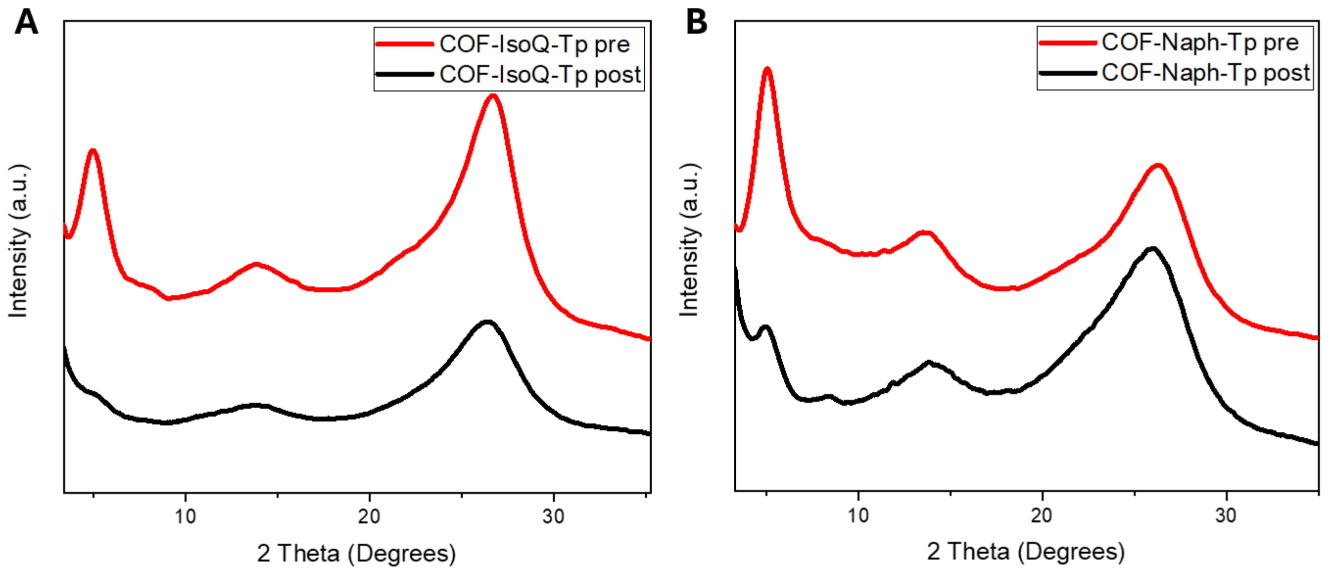

Figure S24. PXRD patterns before (red trace) and after (black trace) photocatalytic experiments for (A) COF-IsoQ-Tp and (B) COF-Naph-Tp.

S3.14: Transmission Electron Microscopy (TEM)

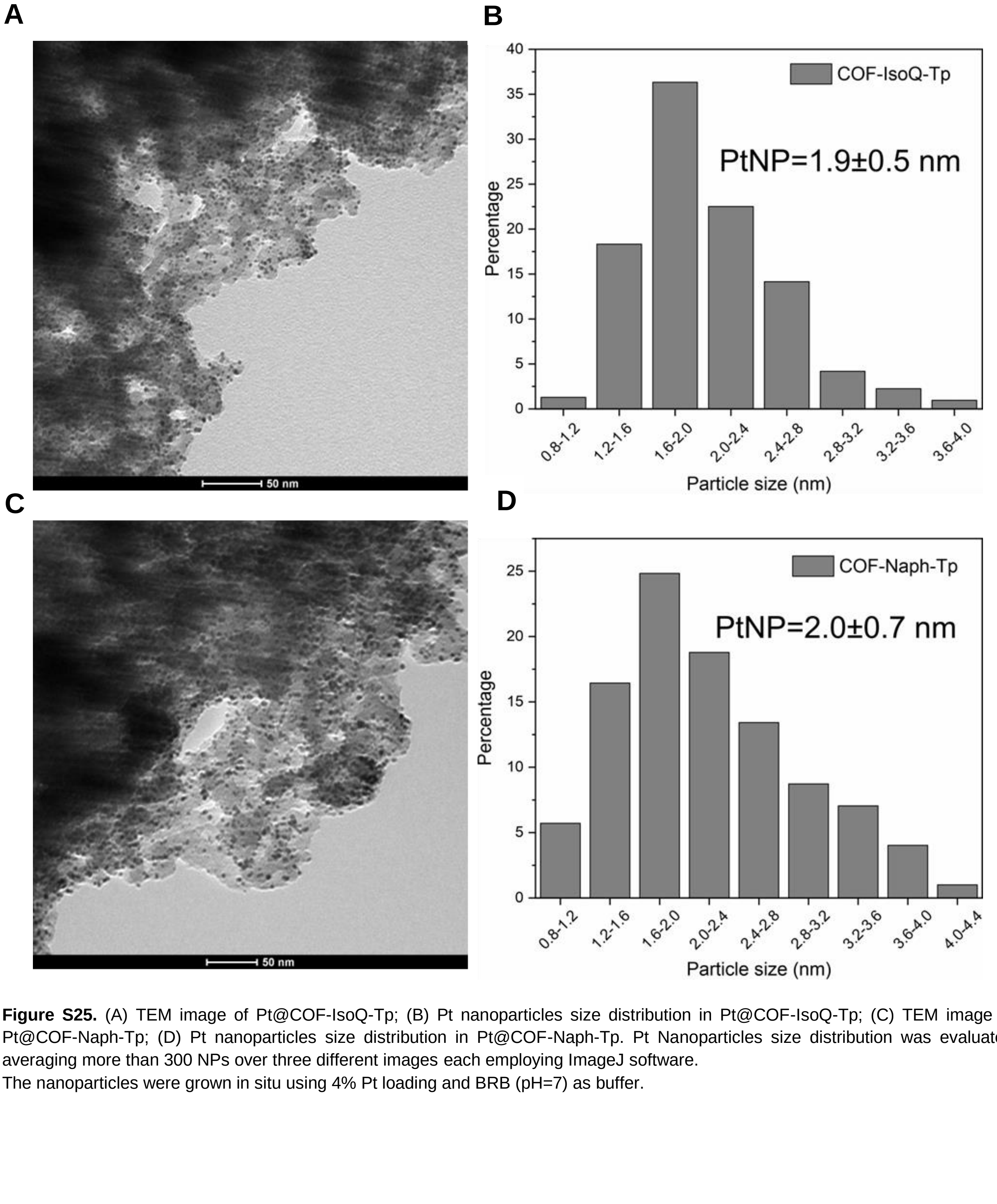

Figure S25. (A) TEM image of Pt@COF-IsoQ-Tp; (B) Pt nanoparticles size distribution in Pt@COF-IsoQ-Tp; (C) TEM image of Pt@COF-Naph-Tp; (D) Pt nanoparticles size distribution in Pt@COF-Naph-Tp. Pt Nanoparticles size distribution was evaluated averaging more than 300 NPs over three different images each employing ImageJ software.
The nanoparticles were grown in situ using 4% Pt loading and BRB (pH=7) as buffer.

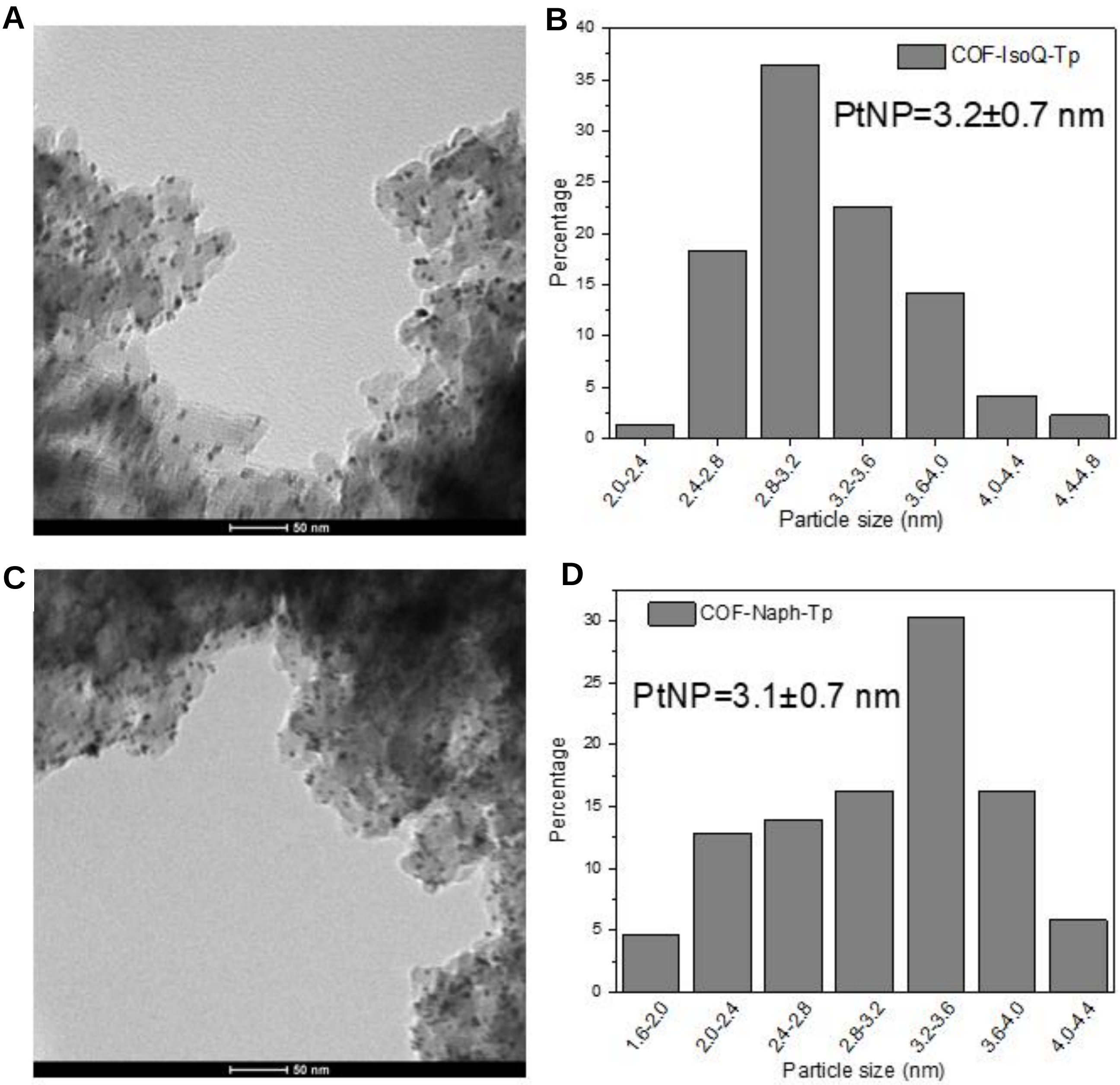

Figure S26. (A) TEM image of Pt@COF-IsoQ-Tp; (B) Pt nanoparticles size distribution in Pt@COF-IsoQ-Tp; (C) TEM image of Pt@COF-Naph-Tp; (D) Pt nanoparticles size distribution in Pt@COF-Naph-Tp. Pt Nanoparticles size distribution was evaluated averaging more than 300 NPs over three different images each employing ImageJ software.
The nanoparticles were grown in situ using 4% Pt loading and BRB (pH=5) as buffer.

S3.15: Pt Loading by ICP-OES

Table S9. % Pt determination by ICP-OES after photocatalytic experiments at various pH values for COF-IsoQ-Tp.

	pH	Theoretical % Pt	Found % Pt
**COF-IsoQ-Tp**	7	4	2.8
**COF-IsoQ-Tp**	5	4	2.8
**COF-IsoQ-Tp**	3	4	3.3

Table S10. Pt determination by ICP-OES after photocatalytic experiments at various pH values for COF-Naph-Tp.

	pH	Theoretical % Pt	Found % Pt
**COF-Naph-Tp**	7	4	1.0
**COF- Naph -Tp**	5	4	1.6
**COF- Naph -Tp**	3	4	2.1

Table S11. Pt determination by ICP-OES after photocatalytic experiments at pH 7 for COF-IsoQ-Tp.

	pH	Theoretical % Pt	Found % Pt
**COF-IsoQ-Tp**	7	0.1	0.10
**COF-IsoQ-Tp**	7	0.25	0.25
**COF-IsoQ-Tp**	7	0.5	0.41
**COF-IsoQ-Tp**	7	1	0.63
**COF-IsoQ-Tp**	7	2	1.49
**COF-IsoQ-Tp**	7	4	3.26

Table S12. Pt determination by ICP-OES after photocatalytic experiments at pH 5 for COF-IsoQ-Tp.

	pH	Theoretical % Pt	Found % Pt
**COF-IsoQ-Tp**	5	1	0.9
**COF-IsoQ-Tp**	5	4	2.8
**COF-IsoQ-Tp**	5	6	3.1
**COF-IsoQ-Tp**	5	8	3.2

S4: Theoretical Calculations

S4.1 COF-IsoQ-Tp and COF-Naph-Tp structural and optoelectronic properties

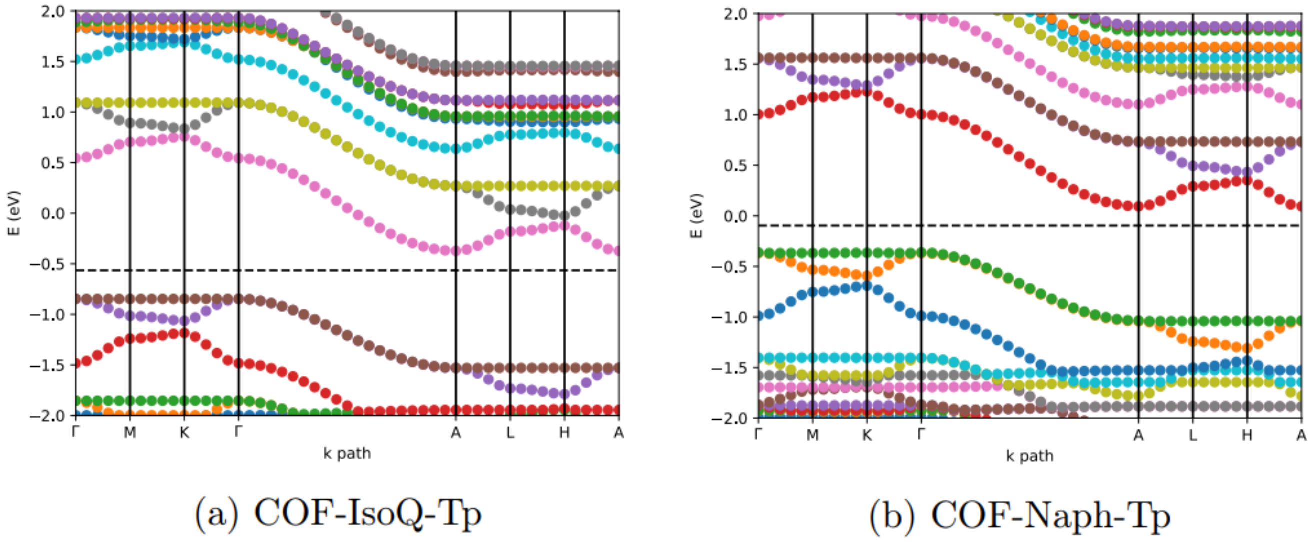

Figure S27. Band structures of (A) COF-IsoQ-Tp and (B) COF-Naph-Tp in a window of 4 eV; the horizontal dashed black line is the Fermi level.

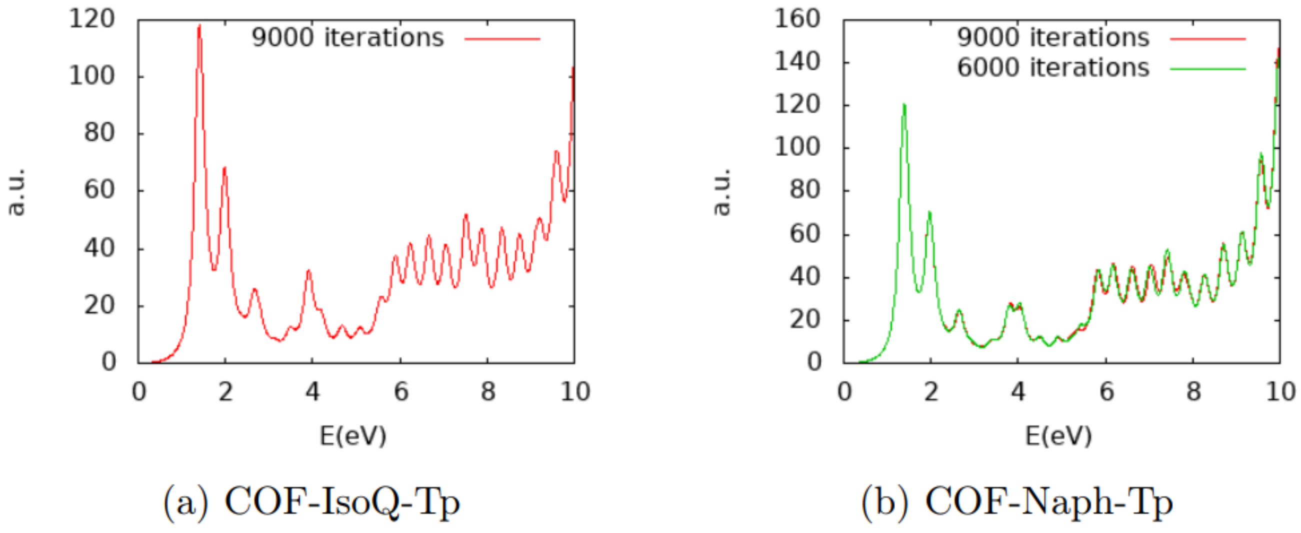

Figure S28. Calculated optical absorption spectra of (A) COF-IsoQ-Tp and (B) COF-Naph-Tp.

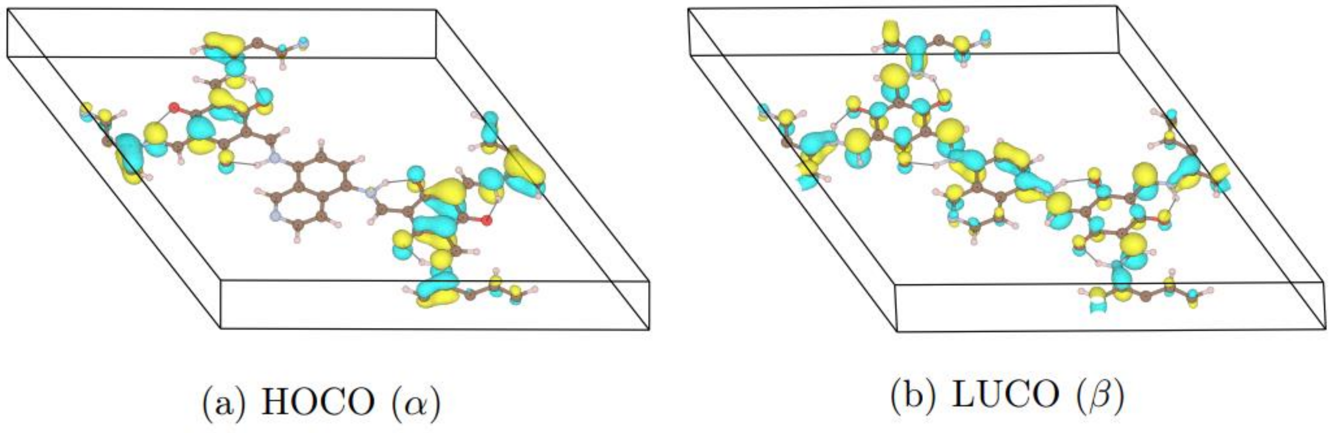

Figure S29. HOCO (α) and LUCO (β) of COF-IsoQ-Tp.

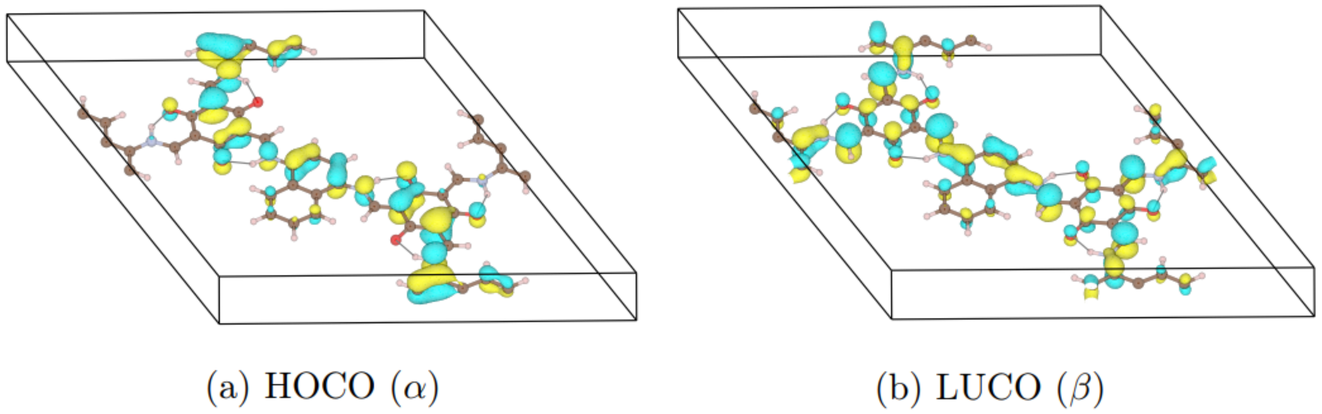

Figure S30. HOCO (α) and LUCO (β) of COF-Naph-Tp.

Computational protocol

All the calculations were performed with the QUANTUM ESPRESSO program package. [29,30] More specifically, the PWscf code was employed for the structural optimization and the band structure calculation of the two COFs, and the turboTDDFT code [31,32] for the calculation of the lowest optical absorption energies. PBE exchange-correlation functional was employed to perform DFT calculations, while the following pseudo-potentials (taken from the QUANTUM ESPRESSO database) were employed to perform electronic structure calculations: H.pbe-kjpaw_psl.1.0.0.UPF for the H atom, C.pbe-nkjpaw_psl.1.0.0.UPF for C, N.pbe-n kjpaw_psl.1.0.0.UPF for N, O.pben-kjpaw_psl.1.0.0.UPF for O and Pt.pbe-n-kjpaw_psl.1.0.0.UPF for Pt. Kinetic energy cutoffs of 100 Ry and 400 Ry were employed for wavefunctions and charge density, respectively.

Computational results

To rationalize the experimental findings provided in this article, a computational characterization of the electronic properties of COF-IsoQ-Tp and COF-Naph-Tp is proposed.

A uniform k-point sampling of 3x3x6 was employed for the optimization of geometries and cell parameters of the two COFs in their eclipsed conformations. The calculated band structure of the two COFs is reported in Figure S27; the bands are plotted along the lines that connect the high symmetry k-points in the following order: Γ → M → K → Γ → A → L → H → A. The band structures reported in Figure S27 are very similar. More specifically, the valence and conduction bands of the two COFs exhibit the same shape. Moreover, the calculated band structures on the planes kz = 0 (Γ → M → K → Γ) and kz = 0.5 (A → L → H → A) are quite similar: this is consistent with the chemical nature of the two investigated systems, which are two-dimensional COFs (i.e. the 3D structure is a stack of sheets of a 2D framework of covalently bounded atoms, with COF sheets interacting through intermolecular forces).[33,34]

The lowest frequencies of the optical absorption spectra were computed at TDDFT level (with the Liouville-Lanczos approach implemented in the turboTDDFT code) for a single k point (the Γ point). The convergence of the results was achieved with 6000 iterations, and the spectra are plotted in Figure S28. A comparison between the experimental and calculated lowest absorption frequencies shows a disagreement of about 0.5 eV for both the COFs (an experimental value equal to 1.9 eV, to be compared with a computational value of 1.4 eV). This is not surprising: calculated band gaps obtained with PBE calculations are known to underestimate the experimental values. On the other hand, the values found for the two COFs are very similar, both at the experimental and at the computational level, suggesting an analogous optical response of the two materials.

S4.2 COF-IsoQ-Tp/Pt_{38} cluster interaction

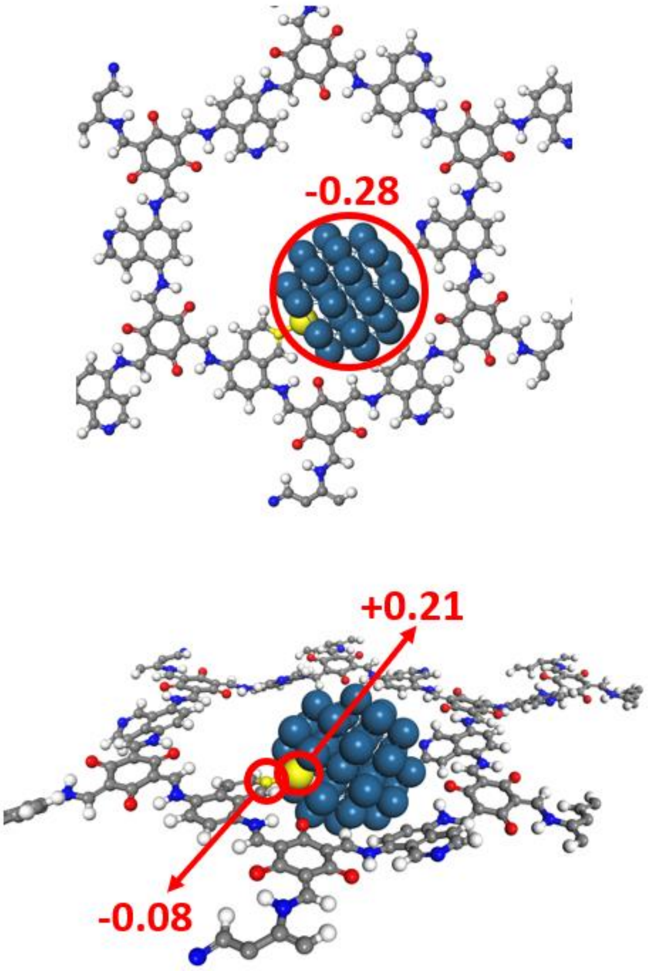

Figure S31. Figure showing the calculated COF-IsoQ-Tp monolayer embedding a platinum nanoparticle composed of 38 atoms. The value in red refers to the charge density obtained by Löwdin population analysis. Overall the Pt_{38} increases its electron density when interacting with the COF backbone while locally the isoquinoline nitrogen acquired electron density when interacting with the central Pt atom of the Pt(111) surface of the nanoparticle.

S5: References

[1] J. Rouquerol, P. Llewellyn, F. Rouquerol, **2007**, pp. 49–56.
[2] J. Jagiello, C. Ania, J. B. Parra, C. Cook, *Carbon N Y* **2015**, *91*, 330–337.
[3] S. L. Murov, I. Carmichael, G. L. Hug, *Handbook of Photochemistry*, CRC Press, **1993**.
[4] J. H. Chong, M. Sauer, B. O. Patrick, M. J. MacLachlan, *Org Lett* **2003**, *5*, 3823–3826.
[5] P. K. Joseph, M. M. Joullié, *J Med Chem* **1964**, *7*, 801–803.
[6] K. T. Potts, D. Bhattacharjee, E. B. Walsh, *J Org Chem* **1986**, *51*, 2011–2021.
[7] L. F. Fieser, *Experiments in Organic Chemistry* , Heath, Boston, MA, **1957**.
[8] J. Ming, A. Liu, J. Zhao, P. Zhang, H. Huang, H. Lin, Z. Xu, X. Zhang, X. Wang, J. Hofkens, M. B. J. Roeffaers, J. Long, *Angewandte Chemie* **2019**, *131*, 18458–18462.
[9] J. L. Sheng, H. Dong, X. Bin Meng, H. L. Tang, Y. H. Yao, D. Q. Liu, L. L. Bai, F. M. Zhang, J. Z. Wei, X. J. Sun, *ChemCatChem* **2019**, *11*, 2313–2319.
[10] T. Banerjee, B. V. Lotsch, *Nat Chem* **2018**, *10*, 1175–1177.
[11] H. Shen, D. Shang, L. Li, D. Li, W. Shi, *Appl Surf Sci* **2022**, *578*, 152024.
[12] M. Wang, Z. Wang, M. Shan, J. Wang, Z. Qiu, J. Song, Z. Li, *Chemistry of Materials* **2023**, *35*, 5368–5377.
[13] G. B. Wang, F. C. Zhu, Q. Q. Lin, J. L. Kan, K. H. Xie, S. Li, Y. Geng, Y. Bin Dong, *Chemical Communications* **2021**, *57*, 4464–4467.
[14] R. Chen, Y. Wang, Y. Ma, A. Mal, X. Y. Gao, L. Gao, L. Qiao, X. B. Li, L. Z. Wu, C. Wang, *Nat Commun* **2021**, *12*, DOI 10.1038/s41467-021-21527-3.
[15] Z. Li, T. Deng, S. Ma, Z. Zhang, G. Wu, J. Wang, Q. Li, H. Xia, S. W. Yang, X. Liu, *J Am Chem Soc* **2022**, DOI 10.1021/jacs.2c11893.
[16] L. Dai, A. Dong, X. Meng, H. Liu, Y. Li, P. Li, B. Wang, *Angewandte Chemie - International Edition* **2023**, *62*, DOI 10.1002/anie.202300224.
[17] S. Xu, H. Sun, M. Addicoat, B. P. Biswal, F. He, S. W. Park, S. Paasch, T. Zhang, W. Sheng, E. Brunner, Y. Hou, M. Richter, X. Feng, *Advanced Materials* **2021**, *33*, DOI 10.1002/adma.202006274.
[18] S. Wei, W. Zhang, P. Qiang, K. Yu, X. Fu, D. Wu, S. Bi, F. Zhang, *J Am Chem Soc* **2019**, *141*, 14272–14279.
[19] J. Xu, C. Yang, S. Bi, W. Wang, Y. He, D. Wu, Q. Liang, X. Wang, F. Zhang, *Angewandte Chemie - International Edition* **2020**, *59*, 23845–23853.
[20] T. Sick, A. G. Hufnagel, J. Kampmann, I. Kondofersky, M. Calik, J. M. Rotter, A. Evans, M. Döblinger, S. Herbert, K. Peters, D. Böhm, P. Knochel, D. D. Medina, D. Fattakhova-Rohlfing, T. Bein, *J Am Chem Soc* **2018**, *140*, 2085–2092.
[21] T. Zhou, L. Wang, X. Huang, J. Unruangsri, H. Zhang, R. Wang, Q. Song, Q. Yang, W. Li, C. Wang, K. Takahashi, H. Xu, J. Guo, *Nat Commun* **2021**, *12*, DOI 10.1038/s41467-021-24179-5.
[22] L. Stegbauer, S. Zech, G. Savasci, T. Banerjee, F. Podjaski, K. Schwinghammer, C. Ochsenfeld, B. V. Lotsch, *Adv Energy Mater* **2018**, *8*, DOI 10.1002/aenm.201703278.
[23] Z. Fu, X. Wang, A. M. Gardner, X. Wang, S. Y. Chong, G. Neri, A. J. Cowan, L. Liu, X. Li, A. Vogel, R. Clowes, M. Bilton, L. Chen, R. S. Sprick, A. I. Cooper, *Chem Sci* **2020**, *11*, 543–550.
[24] S. Li, R. Ma, S. Xu, T. Zheng, G. Fu, Y. Wu, Z. Liao, Y. Kuang, Y. Hou, D. Wang, P. S. Petkov, K. Simeonova, X. Feng, L. Z. Wu, X. B. Li, T. Zhang, *J Am Chem Soc* **2022**, *144*, 13953–13960.
[25] C. Li, J. Liu, H. Li, K. Wu, J. Wang, Q. Yang, *Nat Commun* **2022**, *13*, DOI 10.1038/s41467-022-30035-x.
[26] S. Bag, H. S. Sasmal, S. P. Chaudhary, K. Dey, D. Blätte, R. Guntermann, Y. Zhang, M. Položij, A. Kuc, A. Shelke, R. K. Vijayaraghavan, T. G. Ajithkumar, S. Bhattacharyya, T. Heine, T. Bein, R. Banerjee, *J Am Chem Soc* **2023**, *145*, 1649–1659.
[27] L. Yao, N. Guijarro, F. Boudoire, Y. Liu, A. Rahmanudin, R. A. Wells, A. Sekar, H. H. Cho, J. H. Yum, F. Le Formal, K. Sivula, *J Am Chem Soc* **2020**, *142*, 7795–7802.
[28] Y. Zhang, L. Cao, G. Bai, X. Lan, *Small* **2023**, *19*, DOI 10.1002/smll.202300035.
[29] P. Giannozzi, S. Baroni, N. Bonini, M. Calandra, R. Car, C. Cavazzoni, D. Ceresoli, G. L. Chiarotti, M. Cococcioni, I. Dabo, A. Dal Corso, S. de Gironcoli, S. Fabris, G. Fratesi, R. Gebauer, U. Gerstmann, C. Gougoussis, A. Kokalj, M. Lazzeri, L. Martin-Samos, N. Marzari, F. Mauri, R. Mazzarello, S. Paolini, A. Pasquarello, L. Paulatto, C. Sbraccia, S. Scandolo, G. Sclauzero, A. P. Seitsonen, A. Smogunov, P. Umari, R. M. Wentzcovitch, *Journal of Physics: Condensed Matter* **2009**, *21*, 395502.
[30] P. Giannozzi, O. Andreussi, T. Brumme, O. Bunau, M. Buongiorno Nardelli, M. Calandra, R. Car, C. Cavazzoni, D. Ceresoli, M. Cococcioni, N. Colonna, I. Carnimeo, A. Dal Corso, S. de Gironcoli, P. Delugas, R. A. DiStasio, A. Ferretti, A. Floris, G. Fratesi, G. Fugallo, R. Gebauer, U. Gerstmann, F. Giustino, T. Gorni, J. Jia, M. Kawamura, H.-Y. Ko, A. Kokalj, E. Küçükbenli, M. Lazzeri, M. Marsili, N. Marzari, F. Mauri, N. L. Nguyen, H.-V. Nguyen, A. Otero-de-la-Roza, L. Paulatto, S. Poncé, D. Rocca, R. Sabatini, B. Santra, M. Schlipf, A. P. Seitsonen, A. Smogunov, I. Timrov, T. Thonhauser, P. Umari, N. Vast, X. Wu, S. Baroni, *Journal of Physics: Condensed Matter* **2017**, *29*, 465901.
[31] X. Ge, S. J. Binnie, D. Rocca, R. Gebauer, S. Baroni, *Comput Phys Commun* **2014**, *185*, 2080–2089.
[32] O. B. Malcıoğlu, R. Gebauer, D. Rocca, S. Baroni, *Comput Phys Commun* **2011**, *182*, 1744–1754.
[33] W. Jiang, X. Ni, F. Liu, *Acc Chem Res* **2021**, *54*, 416–426.
[34] X. Ni, H. Li, F. Liu, J.-L. Brédas, *Mater Horiz* **2022**, *9*, 88–98.
